# Multiscale Brazil nut effects in bioturbated sediment

**DOI:** 10.1038/s41598-022-14276-w

**Published:** 2022-07-06

**Authors:** Tatiana Savranskaia, Ramon Egli, Jean-Pierre Valet

**Affiliations:** 1 Institut de Physique Du Globe de Paris, CNRS, Université de Paris, 75005 Paris, France; 2 Zentralanstalt für Meteorologie und Geodynamik (ZAMG), 1190 Wien, Austria; 3 Present Address: Helmholtz-Zentrum Potsdam Deutsches GeoForschungsZentrum GFZ, Telegrafenberg, 14473 Potsdam, Germany

**Keywords:** Sedimentology, Ocean sciences, Stratigraphy

## Abstract

Size segregation in granular materials is a universal phenomenon popularly known as the Brazil nut effect (BNE), from the tendency of larger nuts to end on the top of a shaken container. In nature, fast granular flows bear many similarities with well-studied mixing processes. Instead, much slower phenomena, such as the accumulation of ferromanganese nodules (FN) on the seafloor, have been attributed to the BNE but remain essentially unexplained. Here we document, for the first time, the BNE on sub-millimetre particles in pelagic sediment and propose a size segregation model for the surface mixed layer of bioturbated sediments. Our model explains the size distribution of FN seeds, pointing to a uniform segregation mechanism over sizes ranging from < 1 mm to > 1 cm, which does not depend on selective ingestion by feeding organisms. In addition to explaining FN nucleation, our model has important implications for microfossil dating and the mechanism underlying sedimentary records of the Earth’s magnetic field.

## Introduction

If a container filled with mixed nuts is shaken, size segregation occurs, with the larger Brazil nuts ending on the top^1,2^. This counterintuitive phenomenon is known as the Brazil nut effect (BNE). The BNE appears in processes involving granular mixing or flow^3^. In simple terms, it is caused by the ability of small particles to infiltrate voids that develop preferentially beneath large particles when the interlocking structure of granular materials is disrupted during shaking or shearing^1,4–7^. Despite the simplicity of this principle, the BNE depends in a surprisingly complex manner on how the local structure of granular materials gets disrupted, the material cohesion, and the relative density of the constituents^8^.

The BNE occurs also in geological transport processes: for instance, riverbeds get stabilized by the surface accumulation or large pebbles during bedload transport^9^. Fast dynamics in geophysical mass flows bears many similarities with well-studied industrial granular mixing processes^10,11^. Much slower phenomena, such as the natural lifting of buried archaeological artifacts^12^, the migration of coarse debris to the sediment surface^13^, and the accumulation of ferromanganese nodules (FN) on the seafloor^14^, remain essentially unexplained, despite having been ascribed to a form of bioturbation-driven BNE, in which burrowing organisms push aside particles that are too large to be ingested^13^. The extreme slowness of this ‘biological pumping’ machine prevents a direct observation of the BNE, so that its existence is usually inferred by exclusion of alternate explanations, as it is the case for the relative scarcity of buried FN^15–17^. While FN nuclei^18^ are large enough to remain on the sediment surface and grow, we are left with the question whether objects of the size of microfossils would also be affected by the BNE. This is an important issue for radiocarbon dating, since an upward offset would make them older than the surrounding sediment, as indeed sometimes observed^19,20^, contrary to the negative age offset caused by the preferential dissolution of weaker shells, known as the Barker effect^21^.

**Figure 1:**
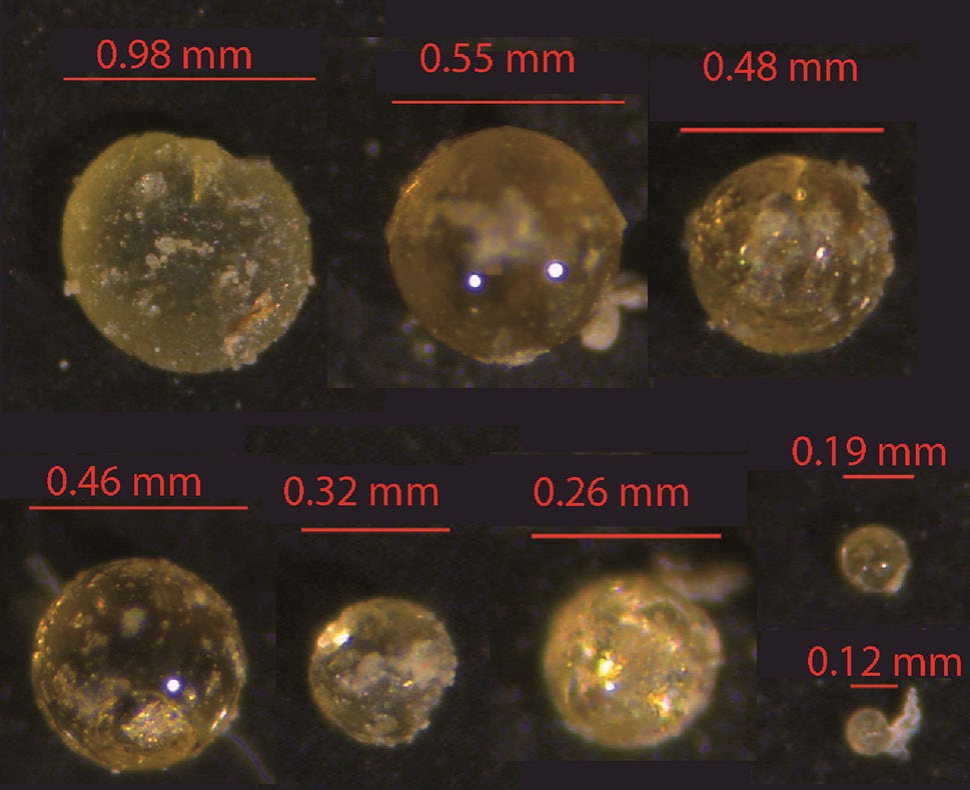
Examples of microtektite fragments found in the Indian Ocean core MD90-0961.

Here we document, for the first time, the BNE occurrence on sub-millimetre microtektite fragments (Fig. 1) that have been deposited ~ 788 ka ago^22^ in a pelagic sediment in the Indian Ocean. Because this was an instantaneous event on the geological timescale, distinct depth distributions for different microtektite size classes represent the impulse response generated by the combined action of sediment mixing and size segregation in the surface mixed layer (SML). The observed impulse responses have been modelled with a size segregation mechanism based on the shear-induced BNE. Our model predicts the correct minimum size of FM nuclei and the microfossil age offsets required to reconcile observed discrepancies.

## Diffusion–advection model of particle segregation

**Figure 2:**
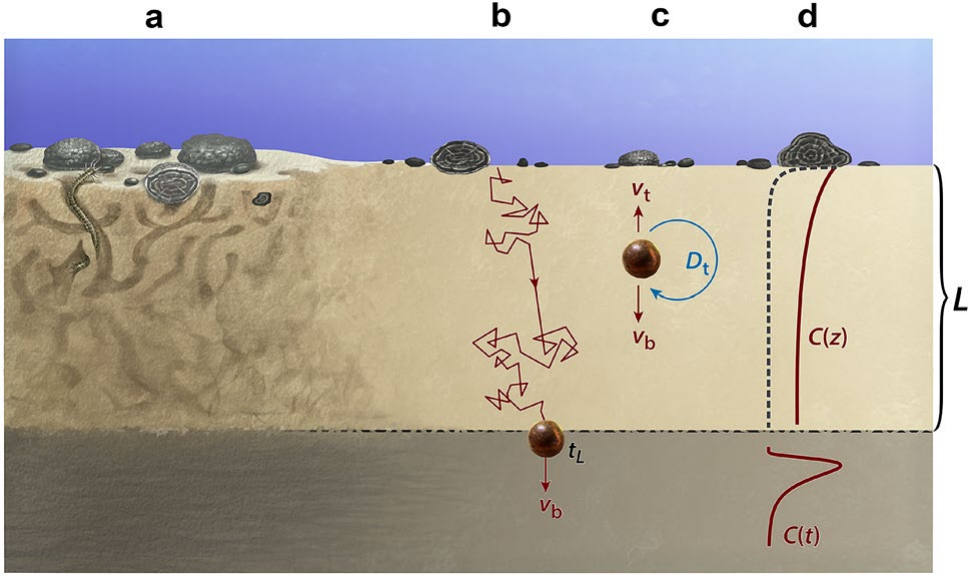
Particle transport within the SML. (**a**) Burrowing and reworking by benthic organisms. (**b**) Individual particles (e.g., a microtektite fragment) perform a biased random walk starting from the sediment surface, until disturbances cease below the SML. (**c**) Under certain conditions, the random walk in (**b**) is governed by a diffusion–advection equation, where the advective velocity is the sum of burial, bioadvective, and size segregation velocities. (**d**) Depth-dependent concentration *C* of tracer particles that are small enough to get buried (e.g., microtektite fragments, solid line) and of ferromanganese nodules large enough to stay indefinitely in the top part or the SML (dashed line). The curve below the SML indicates the microtektite distribution inside the historical layer resulting from an instantaneous deposition process.

Sediment mixing in the SML is caused by bioturbation (Fig. 2a). Mixing is represented mathematically by a stochastic process in which individual sediment particles perform a biased random walk^23^, until they reach the bottom of the SML, where the randomizing action of bioturbation ceases (Fig. 2b). In case of random walks with finite waiting time and jump length distributions^23^, the concentration *C* of a conservative tracer (in our case, microtektites) inside a SML where each volume element undergoes sufficient (i.e., ~ 25) bioturbation events^24^ before being definitively buried, is governed by a simplified version of the diffusion–advection equation obtained by neglecting the porosity gradient^25,26^:

1 $$\frac{\partial C}{\partial t}=\frac{\partial }{\partial z}\left({D}_{\mathrm{t}}\frac{\partial C}{\partial z}\right)-\left({v}_{\mathrm{b}}-{v}_{\mathrm{t}}\right)\frac{\partial C}{\partial z} ,$$

where *t* and *z* are the time and the depth below the sediment–water interface, respectively, ${D}_{\mathrm{t}}$ is the diffusion coefficient of the tracer particles, ${v}_{\mathrm{b}}$ the bulk burial velocity, and ${v}_{\mathrm{t}}$ an additional upward velocity of the tracer particles, due for instance to bio-advection or size segregation (Fig. 2c). The upper boundary condition $\left({v}_{\mathrm{b}}-{v}_{\mathrm{t}}\right)C-{D}_{\mathrm{t}}{\partial }_{z}C={F}_{\mathrm{t}}/{\varphi }_{\mathrm{s}}{\rho }_{\mathrm{s}}$ at *z* = 0, where ${\varphi }_{\mathrm{s}}$ is the volume fraction of solids and ${\rho }_{\mathrm{s}}$ their density, is controlled by the incoming tracer flux ${F}_{\mathrm{t}}$. A microtektite input event is then described by ${F}_{\mathrm{t}}={\Phi }_{\mathrm{t}}\delta (t)$ where $\delta (t)$ is the Dirac impulse, and ${\Phi }_{\mathrm{t}}$ the microtektite fluence.

Bioturbation intensity declines with depth, so that ${D}_{\mathrm{t}}$ and ${v}_{\mathrm{t}}$ are unknown functions of *z*. In practice, different depth-dependent diffusion models yield similar fits to experimental data^25,27^, which means that the SML can be represented by an equivalent homogeneous layer with thickness *L* and constant ${D}_{\mathrm{t}}$, ${v}_{\mathrm{b}}$ and ${v}_{\mathrm{t}}$. Solution of Eq. (1) with $C\left(0,z\right)=\delta (z)$ yields the impulse response $\mathcal{I}\left(t\right)=C(t,L)$ of the system, which can be converted to a depth-dependent concentration profile over $z>L$ using the age model of the sediment (Fig. 2d). The microscopic equivalent to the impulse response is a Wiener process with constant drift, starting at $\left(t,z\right)=(\mathrm{0,0})$ and ending at $\left(t,z\right)=({t}_{L},L)$, where ${t}_{L}$ is the transit (or escape) time^28^ with probability density function $\mathcal{I}\left(t\right)$. The age *T* of particles found at depth $z>L$ is a stochastic variable related to ${t}_{L}$ by $T={t}_{L}+{t}_{\mathrm{b}}$, where ${t}_{\mathrm{b}}$ is the burial time from the bottom of the SML derived from the age model. The stochasticity of *T* is an important factor affecting single specimen dating^29^.

**Figure 3:**
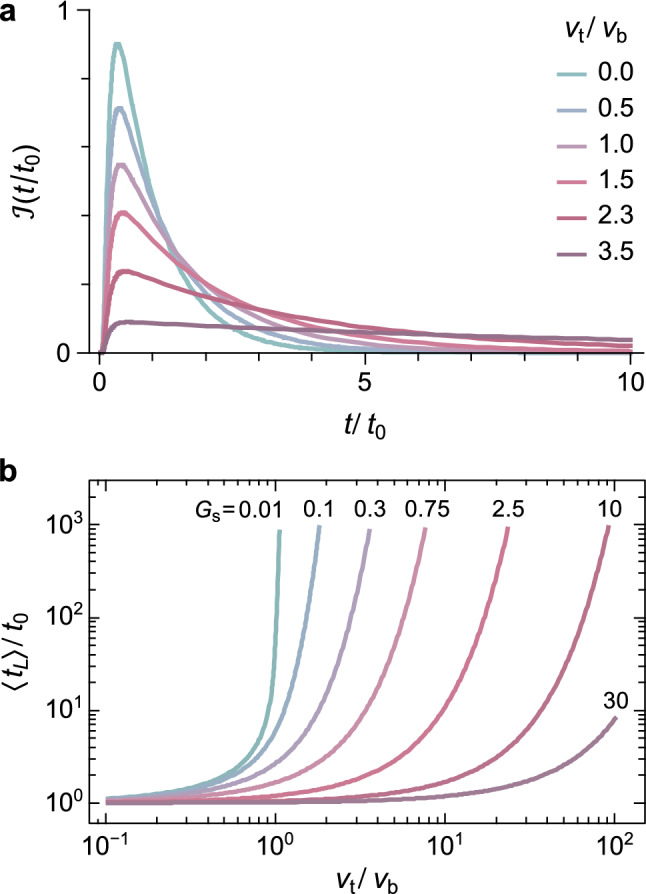
Effect of advection on distribution and age of sediment particles. (**a**) Modelled impulse response of tracer particles, with ${t}_{0}=L/{v}_{\mathrm{b}}$ being the mean transit time of regular sediment particles through the SML, for selected ratios between the tracer segregation velocity ${v}_{\mathrm{t}}$ and the burial velocity ${v}_{\mathrm{b}}$. (**b**) Transit time enhancement as a function of ${v}_{\mathrm{t}}/{v}_{\mathrm{b}}$ for selected values of the inverse Péclet number ${G}_{\mathrm{s}}$ of the bulk sediment.

Solution examples (Fig. 3a) show how ${v}_{\mathrm{t}}$ increases the time needed to cross the SML, due to the reduced or inverted tracer velocity ${v}_{\mathrm{b}}-{v}_{\mathrm{t}}$. The mean transit time $\langle {t}_{L}\rangle$, defined as the expectation of $\mathcal{I}\left(t\right)$, diverges above a critical ${v}_{\mathrm{t}}/{v}_{\mathrm{b}}$ threshold (Fig. 3b). This threshold is close to 1 when advection is the dominant transport mechanism of the tracer particles across the SML. Diffusion ensures a non-negligible probability to escape the SML even if ${v}_{\mathrm{t}}>{v}_{\mathrm{b}}$, yielding a higher ${v}_{\mathrm{t}}/{v}_{\mathrm{b}}$ threshold that depends on the inverse Péclet number $G={D}_{\text{s}}/L{v}_{\mathrm{b}}$ of the bulk sediment, where ${D}_{\mathrm{s}}$ is the bulk diffusion coefficient. In all cases, $\mathcal{I}\left(t\right)$ becomes dramatically skewed as the threshold is approached, converging to a uniform distribution over *t* > 0. This means that size segregation tends to redistribute large particles above the stratigraphic depth corresponding to their deposition age. The grain size dependence of $\mathcal{I}\left(t\right)$ has obvious consequences for dating. While *G* affects the skewness of $\mathcal{I}\left(t\right)$, and thus the stratigraphic age of individual particles, but not the mean age^26^—since $\langle {t}_{L}\rangle =L/{v}_{\mathrm{b}}$ for ${v}_{\mathrm{t}}=0$—size segregation increases the apparent age of larger particles with respect to the bulk, individually and on average, until a meaningful stratigraphic relation is lost.

## Microtektite profiles

The expected concentration of microtektites belonging to a given size interval $[{s}_{1},{s}_{2}]$ is governed by the model function

2 $$C\left(z\right)=\frac{{\Phi }_{\mathrm{t}}}{{\rho }_{\mathrm{s}}{\varphi }_{\mathrm{s}}({z}_{0})}{\int }_{{s}_{1}}^{{s}_{2}}{g}_{\mathrm{t}}\left(s\right)\mathcal{I}\left(\frac{{z}_{0}-z}{{v}_{\mathrm{b}}({z}_{0})};{D}_{\mathrm{t}},{v}_{\mathrm{t}}, L\right)\mathrm{d}s,$$

where ${g}_{\mathrm{t}}$ is the empirical grain size distribution determined from microtektite counts over all depths (Supplementary Figure S1), ${z}_{0}$ the depth in sediment corresponding to the time of the deposition event, and $\mathcal{I}$ the impulse response obtained from the solution of Eq. (1). While ${v}_{\mathrm{b}}$ is derived from the age model of the sediment core, ${\Phi }_{\mathrm{t}}$, ${D}_{\mathrm{t}}$, $L$, and ${v}_{\mathrm{t}}$ must be determined by fitting microtektite profiles for different size classes. Because conventional diffusion–advection models based on ${v}_{\mathrm{t}}=0$ provide good fits of stable tracer concentration profiles^30^, parameter estimates obtained with Eq. (2) cannot be entirely significant, so that additional constraints need to be applied to the size dependencies of ${D}_{\mathrm{t}}$ and ${v}_{\mathrm{t}}$.

Segregation of large particles in the SML can be driven either directly by the BNE, or indirectly by biogenic graded bedding resulting from the selective transport of finer particles through ingestion^31–33^, burrow lining^34^, infills^35^, and resuspension^36^. While graded bedding has been observed in sediments dominated by single benthic organisms, it is not a typical feature of regularly deposited sediment^37,38^. Furthermore, size segregation resulting from graded bedding is not expected to depend on particle size above the maximum dimension of ingestible particles, while, as shown later, this dependence is required to explain the formation of FN. Plastic deformation of sediment around burrowing benthic organisms^39,40^ is a possible BNE driving mechanism, because the deformation field includes a vertical gradient of horizontal displacement around burrow tips, which is analogous to the horizontal shearing used in many BNE experiments^6,41^. In this case, both the diffusivity and the segregation velocity are proportional to the shear rate^6,42^. Size segregation might also be driven by microbial-induced bubble formation in organic-rich sediments^43^.

Experiments with sorted glass beads^44^, which share with microtektites the lack of preferential ingestion by feeding organism^31^, indicate that the size dependence of tracer diffusivity is governed by a power law of the form ${D}_{\mathrm{t}}\propto {s}^{-q}$ with *q* ≈ 0.52. Percolation of smaller particles through random media also displays a power-law dependence on particle size^45^. Accordingly, we assume that the diffusion of large tracer particles with size $s$ in a sediment with mean grain size ${s}_{0}$ is given by ${D}_{\mathrm{t}}={{D}_{\mathrm{s}}(s/{s}_{0})}^{-q}$. Granular mixing experiments show that the advective velocity of large grains is proportional to $s/{s}_{0}-{\psi }_{\mathrm{c}}$, where ${\psi }_{\mathrm{c}}\approx$ 2.8 is a critical size ratio threshold in binary mixtures^4,8^. Therefore, we model the segregation velocity as ${v}_{\mathrm{t}}=(s/{s}_{0}-{\psi }_{\mathrm{c}}){\beta }_{0}{D}_{\mathrm{s}}$, with ${\beta }_{0}$ being an unknown coefficient that expresses the segregation efficiency of bioturbation. Most granular mixing studies have been performed with cohesionless particles, which are a poor analogue to fine-grained sediment. Experiments with wetted grains show that cohesiveness tends to reduce size segregation^46^, as long a clumping is prevented, although this effect is much less pronounced in case of non-spherical particles^47^. Cohesive forces tend to suppress the ability of small particles to infiltrate voids, as they cannot freely fall. However, the biasing effect of gravity, which is the primary cause of size segregation, does not cease. A similar effect has been observed for the torque experienced by magnetic particles in a cohesive, bioturbated sediment, in presence of a weak magnetic field^48,49^. In this case, the resulting magnetic alignment was found to be proportional to the ratio between the magnetic torque and the torques that resist particle rotation. With these considerations in mind, effects of sediment cohesiveness are entirely accounted by ${\beta }_{0}$.

**Figure 4:**
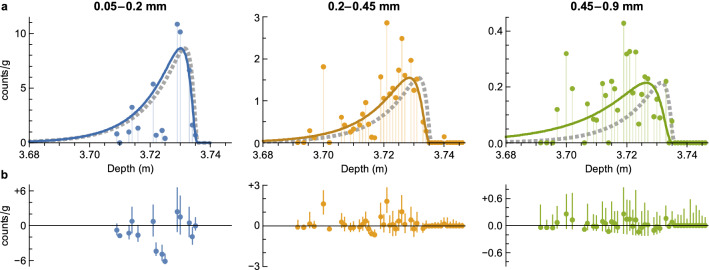
Microtektite distribution fits. (**a**) Concentration of three microtektite size classes in core MD90-0961 (dots), and Poisson fit with Eq. (2) (solid lines). The dashed lines represent the impulse response (rescaled to the same maximum value of the fits) for sediment particles of average size, as predicted by the same model. Notice the offset of large microtektites with respect to the impulse response. (**b**) Model residuals corresponding to the fits in (**a**) (dots), and 90% confidence intervals obtained from a bootstrapping simulation of microtektite counts (error bars).

A Poisson regression model has been used to fit microtektite counts (Fig. 4) for three size classes, using the above models for ${D}_{\mathrm{t}}(s)$ and ${v}_{\mathrm{t}}(s)$. Model residuals are generally compatible with counting uncertainties estimated with bootstrapping, up to few exceptions that might be explained by sediment heterogeneity. The size segregation parameters ${\beta }_{0}$ and *q* are both significantly different from zero at a > 99.4% confidence level (Table 1). Estimates of ${D}_{\mathrm{s}}$ and *L* are comprised within the typical ranges obtained from radioactive tracers for similar sediments^50^. The power-law exponent *q* ≈ 0.25 for the size dependence of ${D}_{\mathrm{s}}$ (Table 1) is smaller than the value obtained by Wheatcroft^44^ for 10–300 µm glass beads, possibly because most microtektites are too large to be ingested.

**Table 1:** Poisson regression models for two null hypotheses ${H}_{0}$ (fixed parameters in parentheses) and the size segregation hypothesis ${H}_{1}$.

Parameter	*H*_0_ (${\beta }_{0}$ = *q* = 0)	*H*_0_ (${\beta }_{0}$ = 0)	*H*_1_ (${\beta }_{0}$ > 0, *q* > 0)
${\Phi }_{\mathrm{t}}$ (counts/g)	288.7	286.1	282.4 ± 19
${z}_{0}$ (cm)	3736.3	3736.3	3736.5 ± 0.9
$L$ (cm)	23.6	23.54	19.2 ± 2
${D}_{\mathrm{s}}$ (cm^2^/kyr)	51.6	122.2	55.7 ± 18
$q$ (–)	(0)	0.327	0.252 ± 0.08
${\beta }_{0}$ (m^–1^)	(0)	(0)	0.075 ± 0.03
*p *value	0.99989	0.9947	–

The *p* value represents the rejection probability of ${H}_{0}={H}_{1}$, obtained from the test statistics $-2 {\text{ln}}\Lambda$ of the likelihood-ratio test. Error estimates for ${H}_{1}$ parameters correspond to the standard deviation of bootstrapped simulation of microtektite counts. Fixed parameters derived from the age model and from physical properties are ${v}_{\mathrm{b}}=$ 3.8 cm/kyr, ${\varphi }_{\mathrm{s}}=$ 0.6, ${\rho }_{\mathrm{s}}=$ 2.6 g/cm^3^, ${s}_{0}=$ 10 µm, ${\psi }_{\mathrm{c}}=$ 3, and ${\varphi }_{\mathrm{s}}({z}_{0})/{\varphi }_{\mathrm{s}}(L)=$ 1.33.

## Stratigraphic, environmental, and paleomagnetic implications

Tektite profiles illustrate how size segregation offsets the age distribution of buried objects (solid lines in Fig. 4a), relative to that of regular sediment particles (dashed lines in Fig. 4a). The predicted mean age offsets for the three size classes of Fig. 4 (~ 0.16 kyr for 0.05–0.2 mm, ~ 0.87 kyr for 0.2–0.45 mm, and ~ 2.9 kyr for 0.45–0.9 mm) are comparable with the maximum positive offsets reported for radiocarbon ages^20,21^. The role of size segregation in the generation of positive age offsets increases with the Péclet number, producing a ‘runaway’ effect when the net burial velocity ${v}_{\mathrm{b}}-{v}_{\mathrm{t}}$ in the SML vanishes (Fig. 3). For this reason, sediments with low deposition rates are expected to be particularly prone to age offsets caused by size segregation. For instance, decreasing ${v}_{\mathrm{b}}$ to 1.5 cm/kyr for a sediment with same properties as MD90-0961 would increase the age offset of a 0.5 mm object from ~ 0.73 to ~ 10 kyr. Large positive offsets caused by the BNE can explain foraminifera age differences that cannot arise from selective dissolution alone^21,51,52^. On the other hand, size segregation is less sensitive to changes of the diffusion coefficient: for instance, doubling ${D}_{\mathrm{s}}$ increases the age offset of the 0.5 mm object of the above example to ~ 1.7 kyr, because the resulting increase of ${v}_{\mathrm{t}}$ is partially compensated by a decrease of Pe.

In case of a stationary flux of large tracer particles, the BNE produces a concentration gradient within the SML with a similar dependence on ${v}_{\mathrm{t}}/{v}_{\mathrm{b}}$ as $\langle {t}_{L}\rangle$ (Supplementary Figure S2): this is because conservation of the vertical tracer flux requires a decrease of the net burial velocity to be compensated by a higher concentration. Hence, the interpretation of foraminiferal concentration variations within the SML might be biased by the BNE. For instance, the concentration of *G. bulloides* tests in sediments of the Oman margin^53,54^, which have been used to reconstruct the Indian summer monsoon during the last ~ 2000 years, might increase by 9–40% in the uppermost ~ 6 cm, if the same segregation parameters of Table 1 are assumed along with bioturbation data representative of the oxygen minimum zone in the northwest Arabian Sea (i.e., ${D}_{\mathrm{s}}\approx$ 150 cm^2^/kyr, *L* ≈ 6 cm, and ${v}_{\mathrm{b}}=$ 3–20 cm/kyr^54,55^).

Size segregation at sub-millimetre scales has important paleomagnetic implications because it requires a reorganization of the sediment microstructure assimilable with a truly diffusive process, which causes a reorientation of magnetic carriers. While non-local transport in the SML is hardly distinguishable from true diffusion^23^, the two processes affect sedimentary records of the Earth magnetic field in a drastically different manner. Upward conveyor belt transport removes material at depth and redeposits it on the sediment surface, where a so-called detrital remanent magnetization (DRM) is acquired by partial alignment of suspended particles in the magnetic field. Buried sediment, which is not affected by bioturbation in this model, would carry an intact DRM coeval with deposition age^56^. Local disruption of the sediment structure, on the other hand, erases the existing DRM and replaces it with a post-depositional magnetization (PDRM) younger than the age of deposition. Conventional PDRM models assume that this magnetization is acquired below the SML during early diagenesis^57^; however, laboratory experiments have shown that PDRM acquisition can be driven by bioturbation through the rotational component of diffusion^48,49^. Diffusive sediment mixing introduces a delay of the order of $L/{v}_{\text{b}}$ in magnetostratigraphic records not affected by diagenesis. This delay is compatible with observed offsets between magnetic mineral and ^10^Be records of the Matuyama-Brunhes field reversal^58^, if appropriated estimates of *L* for marine sediments^50^ are used.

## Implications for ferromanganese nodules

**Figure 5:**
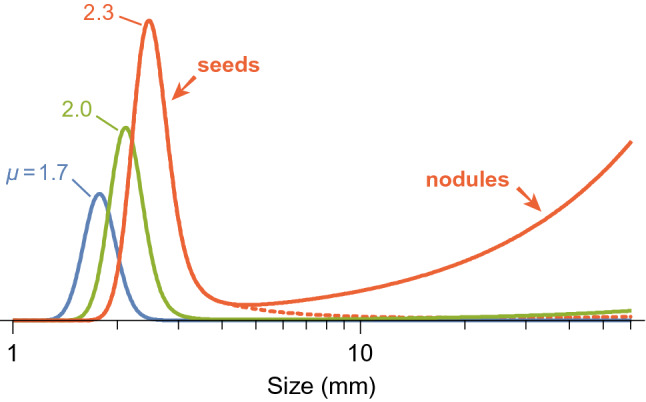
Steady-state distribution of growing ferromanganese nodule seeds predicted by Eq. (3) with size segregation parameters from microtektite fits of core MD90-0961 (Table 1), a nodule grow rate of 5 mm/Myr, and *L* = 6 cm, ${D}_{\mathrm{s}}$ = 22 cm^2^/kyr and ${v}_{\mathrm{b}}$ = 0.5 cm/kyr as representative SML properties at a water depth of ~ 4000 m. Results are shown for lognormal size distributions of depositing seeds (solid lines) with *σ* = 0.1 and three values of *µ* (numbers in mm). The dashed line represents the case of *µ* = 2.3 mm when the linear increase of ${v}_{\mathrm{t}}(s)$ stops at *s* = 4 mm.

If FN nucleation and microtektite segregation have the same origin, segregation parameters estimated from microtektite profiles can be used to predict the minimum size of FN nuclei, which is 1–5 mm^18^. A simple growth model assumes that the size $s\left(T\right)={s}_{\mathrm{n}}+\gamma T$ of a FN of age $T$ increases linearly in time from the initial nucleus size ${s}_{\mathrm{n}}$ at a constant growth rate $\gamma \approx$ 1–5 mm/Myr^59^. In case of a stationary flux ${F}_{0}$ of seeds with initial grain size distribution ${n}_{0}(s)$, the size distribution $n(s)$ of growing seeds at the sediment–water interface is given by

3 $$0=\frac{\partial n}{\partial t}={F}_{0}{n}_{0}-\gamma L\frac{\partial n}{\partial s}-{v}_{\text{b}}rn,$$

where $r(s)$ is the ratio between the tracer concentrations at *z* = *L* and *z* = 0, respectively, obtained from the steady-state solution of Eq. (1). Solutions of Eq. (3) with the size segregation parameters of Table 1 and SML properties typical of FN fields at a water depth of ~ 4000 m^50,60^ predict minimum seed sizes of ~ 2–3 mm (Fig. 5), which are comparable with observed ones^18^. Under these conditions, an increase of ${v}_{\mathrm{b}}$ from 0.5 to 0.8 cm/kyr, a decrease of ${D}_{\mathrm{s}}$ from 22 to 8 cm^2^/kyr, or a decrease of ${\beta }_{0}$ from 0.075 to 0.064 m^−1^ are sufficient to suppress the growth of ~ 2.3 mm seeds, confirming that a minimum degree of bioturbation and a sufficiently small sedimentation rate are necessary physical conditions for FN growth^17^. Finally, a linear dependence of ${v}_{\mathrm{t}}$ on size up to at least ~ 4 mm, well above the limit of selective transport by benthic organisms, is required to reduce the sinking probability of growing FN nuclei so that they can grow by several cm (dashed line in Fig. 5). This demonstrates that FN are kept at the sediment surface by the BNE, rather than other mechanisms like the selective removal of fine-grained sediment by bottom currents^17^.

## Conclusions

We report, for the first time, the size segregation of 0.05–0.9 mm microtektite fragments in a pelagic sediment from the Indian Ocean. The depth distribution of these fragments can be explained by a bioturbation-driven BNE in the SML. As a result, large particles experience an upwardly directed segregation velocity ${v}_{\mathrm{t}}$ relative to the bulk sediment, which increases linearly with particle size. Above a sediment-dependent size threshold (e.g., ~ 1 mm in core MD90-0961), ${v}_{\mathrm{t}}$ exceeds the burial velocity, and the probability of burial below the SML becomes small. This has two important consequences: (1) buried objects that are much larger than the mean grain size of sediment, such as microfossils, are significantly older than their stratigraphic age and tend to lose any relation with stratigraphy for sizes above the ${v}_{\mathrm{t}}={v}_{\mathrm{b}}$ threshold, and (2) > 1 mm particles tend to remain on the sediment surface for long times, serving as seeds for the growth of FN under favourable conditions. In the latter case, continuous growth further decreases the burial probability, explaining the scarcity of buried nodules. A single empirical model for the size dependence of segregation velocity and diffusivity, derived from experiments on granular mixing, explains our microtektite counting results and correctly predicts the minimum size of FN seeds, despite the > 10 orders of magnitude difference between bioturbation and laboratory time scales. Plastic deformation of sediment associated with burrowing is the most likely BNE driving mechanism.

The BNE has important implications for the fundamental understanding of phenomena that depends on sediment micromechanics, such as FN growth and paleomagnetic records, and for the interpretation of foraminifera ages and concentration variations. Large positive age offsets caused by the BNE can explain foraminifera age differences that cannot arise from selective dissolution alone. Furthermore, the BNE produces a concentration gradient within the SML, which might affect the interpretation of recent climatic variations. The effect of physical sediment properties like cohesiveness on size segregation needs to be investigated to assess the role played by the BNE in the redistribution of large particles in sediment, beyond the single example presented here.

## Methods

### Microtektite counting

Core MD90-0961 (5°03.71′ N, 73°52.57′ E) was collected during the SEYMAMA research cruise of the R/V Marion Dufresne in 1990. The 45-m long core was retrieved on the eastern margin of the Chagos-Maldive-Laccadive Ridge at a water depth of 2450 m and is composed of calcareous nannofossil ooze with abundant foraminifera. Typical microtektite concentrations amount to few counts per sample (~ 3 g); therefore, counts from three sampling campaigns (Supplementary Tables S1, S2, S3) have been gathered into three size classes with a total of 137, 287, and 49 counts, respectively (Table 2). Sediment preparation details are given in the Supplementary Information.

**Table 2:** Core MD90-0961 total microtektite counts, regrouped into three size classes.

Core depth (cm)	Mass (g)	0.05–0.20 mm (counts)	Mass (g)	0.20–0.45 mm (counts)	Mass (g)	0.45–0.90 mm (counts)
3691.5			2.02	0	2.02	0
3693.5			6.0548	0	6.0548	0
3695.5			3.53	1	3.53	0
3697			8.36	1	8.36	1
3700			9.386	17	9.386	3
3702			5.15	0	5.15	1
3706			9.89	6	9.89	0
3707			7.167	3	7.167	1
3709	3.7	3	11.7946	3	11.7946	1
3710	3.46	0	12.34	4	12.34	1
3712			9.412	4	9.412	2
3713	3.02	3	11.79	8	11.79	2
3714	2.46	8	10.44	10	10.44	0
3715			9.09	4	9.09	2
3716	2.97	4	15.49	2	15.49	3
3717			8.55	1	8.55	1
3719			6.997	11	6.997	3
3720			9.43	10	9.43	3
3721	2.79	15	12.2387	35	12.2387	4
3722	2.88	2	11.17	13	11.17	2
3723			6.15	8	6.15	2
3724	2.646	3	14.883	16	14.883	1
3725	2.474	1	11.54	20	11.54	2
3726			8.448	21	8.448	2
3727			13.06	21	13.06	3
3729	2.947	32	10.686	21	10.686	1
3730	3.35	34	12.817	16	12.817	2
3731			11.21	17	11.21	1
3733	3.765	25	18.1947	9	18.1947	4
3734	3.092	5	14.5748	4	14.5748	0
3735	2.838	2	12.8858	0	12.8858	1
3736			6.917	0	6.917	0
3737			4.35	0	4.35	0
3738			4.4755	0	4.4755	0
3739			2.7476	0	2.7476	0
3740			7.067	1	7.067	0
3741			1.75	0	1.75	0
3742			3.625	0	3.625	0
3743			2.2875	0	2.2875	0
3744			3.01	0	3.01	0
3745			4.858	0	4.858	0
3746			5.63	0	5.63	0

### Proof of Equations 1–3

The diffusion–advection equation does not have a simple analytical solution in case of size segregation. A series expansion of of $\mathcal{I}\left(t\right)$ was obtained with the solution approach of Guinasso and Schink^26^, taking the modified boundary conditions into account (Supplementary Methods).

### Significance tests

The significance of the size segregation parameters ${\beta }_{0}$ and $q$ have been tested using the ratio $\Lambda$ between the likelihoods of the null hypothesis ${H}_{0}$ that segregation does not occur (${\beta }_{0}$ = 0 or ${\beta }_{0}$ = *q* = 0), and of the full model ${H}_{1}$, assuming that microtektite counts are governed by Poisson statistics (Supplementary Methods).

## Supplementary Information

Supplementary Information.

## Data Availability

All data supporting our findings are given in the Supplementary Tables.
