# Supplementary material for: Multiscale Brazil nut effects in bioturbated sediment
Source: Sci Rep. 2022 Jul 6;12:11450. doi: 10.1038/s41598-022-14276-w (PMC9259689; doi:10.1038/s41598-022-14276-w)
Supplement: Supplementary file 1 — Supplementary Information. [file 41598_2022_14276_MOESM1_ESM.pdf]

Multiscale Brazil nut effects in bioturbated sediment

T. Savranskaia[1]*†, R. Egli[1,2]*, J.-P. Valet[1]

[1]Institut de Physique Du Globe de Paris, CNRS, Université de Paris, 75005, Paris, France

[2]Zentralanstalt für Meteorologie und Geodynamik (ZAMG), 1190 Wien, Austria

*Corresponding authors: tatiana.savranskaia@gfz-potsdam.de, ramon.egli@zamg.ac.at

†Present address: Helmholtz-Zentrum Potsdam, Deutsches GeoForschungsZenutrum GFZ, Telegrafenberg, 14473 Potsdam, Germany

Supplementary Information

Contents:

1. Supplementary Figures and Tables

2. Supplementary Methods

Supplementary Figures and Tables

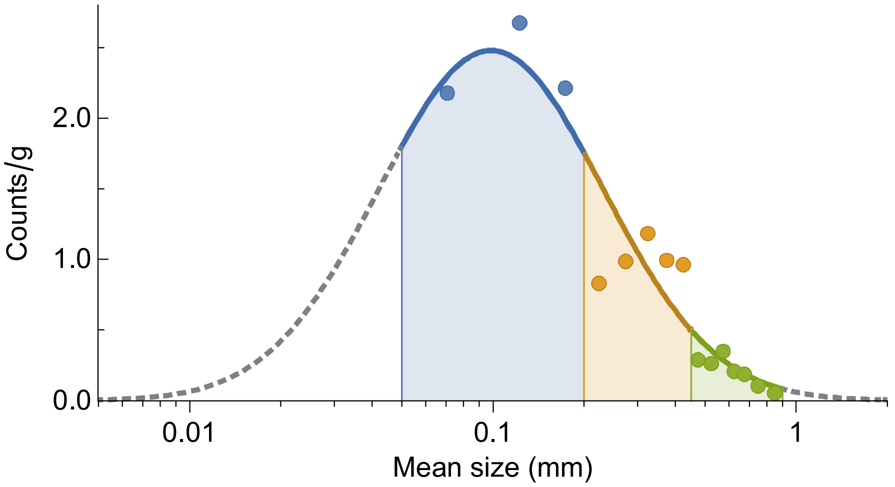

Supplementary Figure S1. Total microtektite counts (dots) for the 15 size classes used in the original counting campaigns (Tables S1-S3), and least-squares fit with a lognormal size distribution (line). Shaded areas represent the total contributions to the 0.05–0.2 mm, 0.2–0.45, and 0.49–0.9 mm size classes used for modeling.

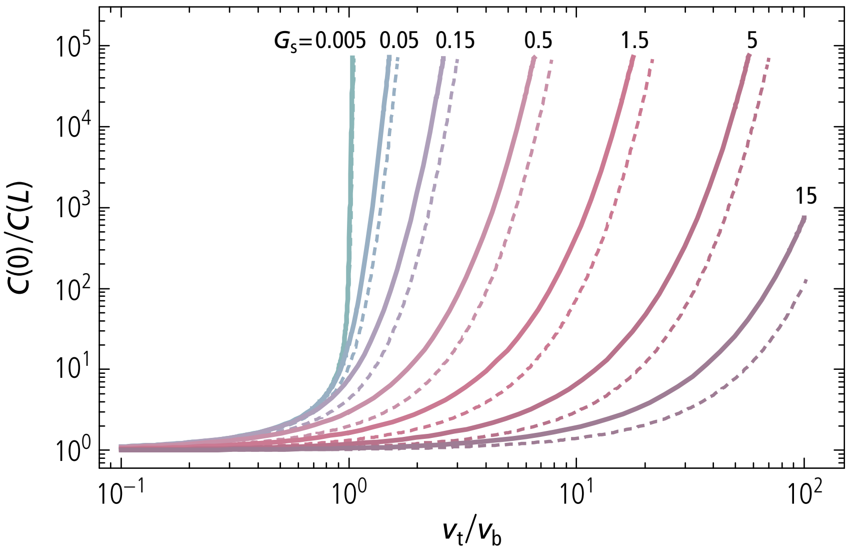

Supplementary Figure S2. Ratio between the tracer concentration at the sediment-water interface and at the bottom of the mixed layer, as a function of v_t/v_b, for selected values of the inverse Péclet number G_s (solid lines). The normalized escape time expectation of Figure 3b in the Article is shown for comparison (dashed lines). The values of G_s correspond to a mixed layer with thickness $L = 10$ cm, burial velocity $v_b = 2$ cm/kyr, and bulk diffusion coefficients $D_s = 0.1$, 1, 3, 10, 30, 100, and 300 cm^2/kyr, respectively. Notice the logarithmic scale on the vertical axis.

Supplementary Table S1. Microtektite counts from the first sampling campaign, divided into 15 size classes.

Core depth (cm)	Mass (g)	0.05–0.10 (cnt)	0.10–0.15 (cnt)	0.15–0.20 (cnt)	0.20–0.25 (cnt)	0.25–0.30 (cnt)	0.30–0.35 (cnt)	0.35–0.40 (cnt)	0.40–0.45 (cnt)	0.45–0.50 (cnt)	0.50–0.55 (cnt)	0.55–0.60 (cnt)	0.60–0.65 (cnt)	0.65–0.70 (cnt)	0.70–0.80 (cnt)	0.80–0.90 (cnt)
3709.0	3.700	1	2	0	0	0	1	0	0	0	0	0	0	0	0	0
3710.0	3.460	0	0	0	0	0	0	0	0	0	0	0	0	0	0	0
3713.0	3.020	1	2	0	0	1	0	0	1	0	0	0	0	0	0	0
3714.0	2.460	4	3	1	0	3	1	0	0	0	0	0	0	0	0	0
3716.0	2.970	1	2	1	0	1	0	0	0	0	0	0	0	0	0	0
3721.0	2.790	4	8	3	4	5	1	0	0	1	1	0	0	1	0	0
3722.0	2.880	0	0	2	1	1	1	1	0	0	0	0	0	0	0	0
3724.0	2.646	0	1	2	0	2	0	1	1	0	0	0	0	0	0	0
3725.0	2.474	0	0	1	3	1	1	0	1	0	0	0	0	0	0	0
3729.0	2.947	11	12	9	3	2	0	0	0	0	0	1	0	0	0	0
3730.0	3.350	15	12	7	3	1	0	2	0	0	0	1	0	0	1	0
3733.0	3.765	22	2	1	3	1	0	0	1	0	0	0	1	0	1	0
3734.0	3.092	4	1	0	0	0	0	0	0	0	0	0	0	0	0	0
3735.0	2.838	1	1	0	0	0	0	0	0	0	0	0	0	0	0	0

Supplementary Table S2. Microtektite counts from the second sampling campaign, divided into 15 size classes.

Core depth (cm)	Mass (g)	0.05–0.10 (cnt)	0.10–0.15 (cnt)	0.15–0.20 (cnt)	0.20–0.25 (cnt)	0.25–0.30 (cnt)	0.30–0.35 (cnt)	0.35–0.40 (cnt)	0.40–0.45 (cnt)	0.45–0.50 (cnt)	0.50–0.55 (cnt)	0.55–0.60 (cnt)	0.60–0.65 (cnt)	0.65–0.70 (cnt)	0.70–0.80 (cnt)	0.80–0.90 (cnt)
3697.0	4.500	0	0	0	0	0	0	0	0	0	0	0	0	0	0	0
3700.0	4.260	0	0	0	0	1	0	0	0	0	0	1	0	0	0	0
3702.0	5.150	0	0	0	0	0	0	0	0	0	0	0	0	0	1	0
3706.0	4.110	0	0	0	0	0	0	0	0	0	0	0	0	0	0	0
3707.0	4.820	0	0	0	0	0	0	0	1	0	0	0	0	0	0	0
3709.0	4.280	0	0	0	0	0	0	0	0	0	0	0	0	0	0	0
3710.0	5.410	0	0	0	0	0	0	0	0	0	0	0	0	0	0	0
3712.0	4.430	0	0	0	0	0	0	1	1	0	0	0	0	0	0	1
3713.0	4.870	0	0	0	0	1	0	0	0	0	0	1	0	0	0	0
3714.0	4.700	0	0	0	0	0	1	0	1	0	0	0	0	0	0	0
3715.0	5.300	0	0	0	1	1	0	0	0	0	0	0	0	0	0	0
3716.0	5.500	0	0	0	1	0	0	0	0	0	0	0	0	1	0	0
3717.0	5.760	0	0	0	0	0	0	0	0	0	0	0	0	0	0	0
3719.0	4.960	0	0	0	1	5	2	0	0	0	0	1	0	0	0	0
3720.0	5.700	0	0	0	1	2	1	1	1	0	0	0	1	1	0	0
3721.0	6.450	0	0	3	1	3	7	2	1	0	0	0	0	0	0	0
3722.0	4.940	0	0	0	3	0	0	1	0	0	0	0	0	0	0	0
3723.0	4.270	0	0	0	0	0	0	2	1	0	0	0	0	0	0	0
3724.0	6.300	0	0	0	1	3	2	3	0	0	0	1	0	0	0	0
3725.0	5.630	0	0	1	0	5	4	0	3	1	0	0	0	0	0	0
3726.0	5.540	0	0	1	2	4	1	5	4	0	1	0	0	1	0	0
3727.0	6.560	0	0	2	3	1	7	0	1	1	0	1	1	0	0	0
3729.0	4.750	0	0	1	2	0	1	3	0	0	0	0	0	0	0	0
3730.0	5.620	0	0	0	0	2	2	1	1	0	0	0	0	0	0	0
3731.0	8.690	0	0	0	5	3	1	3	4	0	1	0	0	0	0	0
3733.0	5.720	0	0	1	2	1	0	0	0	0	0	0	0	0	0	0
3734.0	7.130	0	0	1	0	1	1	1	0	0	0	0	0	0	0	0
3735.0	7.290	0	0	0	0	0	0	0	0	1	0	0	0	0	0	0

Supplementary Table S3. Microtektite counts from the third sampling campaign, divided into 15 size classes.

Core depth (cm)	Mass (g)	0.05–0.10 (cnt)	0.10–0.15 (cnt)	0.15–0.20 (cnt)	0.20–0.25 (cnt)	0.25–0.30 (cnt)	0.30–0.35 (cnt)	0.35–0.40 (cnt)	0.40–0.45 (cnt)	0.45–0.50 (cnt)	0.50–0.55 (cnt)	0.55–0.60 (cnt)	0.60–0.65 (cnt)	0.65–0.70 (cnt)	0.70–0.80 (cnt)	0.80–0.90 (cnt)
3691.5	2.020	0	0	0	0	0	0	0	0	0	0	0	0	0	0	0
3693.5	6.055	0	0	0	0	0	0	0	0	0	0	0	0	0	0	0
3695.5	3.530	0	0	0	1	0	0	0	0	0	0	0	0	0	0	0
3697.0	3.860	0	0	0	0	0	0	0	1	0	0	0	0	0	1	0
3700.0	5.126	0	0	0	3	1	8	4	0	0	0	1	0	0	1	0
3706.0	5.780	0	0	0	2	0	3	0	1	0	0	0	0	0	0	0
3707.0	2.347	0	0	0	0	1	0	1	0	1	0	0	0	0	0	0
3709.0	3.815	0	0	0	0	0	0	1	1	0	0	0	1	0	0	0
3710.0	3.470	0	0	0	1	1	1	0	1	0	1	0	0	0	0	0
3712.0	4.982	0	0	0	0	0	0	1	1	0	0	0	0	1	0	0
3713.0	3.900	0	0	0	0	1	1	1	2	1	0	0	0	0	0	0
3714.0	3.280	0	0	0	2	1	1	0	0	0	0	0	0	0	0	0
3715.0	3.790	0	0	0	1	0	0	0	1	0	1	0	1	0	0	0
3716.0	7.020	0	0	0	0	0	0	0	0	0	1	0	1	0	0	0
3717.0	2.790	0	0	0	0	0	0	0	1	0	1	0	0	0	0	0
3719.0	2.037	0	0	0	0	2	0	1	0	2	0	0	0	0	0	0
3720.0	3.730	0	0	0	0	0	0	1	3	0	1	0	0	0	0	0
3721.0	2.999	0	0	0	1	0	4	2	0	0	0	0	0	0	0	1
3722.0	3.350	0	0	0	0	2	2	0	1	1	1	0	0	0	0	0
3723.0	1.880	0	0	0	1	0	0	3	1	0	0	2	0	0	0	0
3724.0	5.937	0	0	0	0	1	0	1	1	0	0	0	0	0	0	0
3725.0	3.436	0	0	0	0	0	0	1	0	1	0	0	0	0	0	0
3726.0	2.908	0	0	0	2	0	1	0	1	0	0	0	0	0	0	0
3727.0	6.500	0	0	0	1	3	2	1	0	0	0	0	0	0	0	0
3729.0	2.989	0	1	1	0	1	4	1	2	0	0	0	0	0	0	0
3730.0	3.847	0	0	0	0	0	3	1	0	0	0	0	0	0	0	0
3731.0	2.520	0	0	0	0	0	1	0	0	0	0	0	0	0	0	0
3733.0	8.710	0	0	0	0	0	0	0	0	1	0	1	0	0	0	0

Supplementary Table S3. Continued.

Core depth (cm)	Mass (g)	0.05–0.10 (cnt)	0.10–0.15 (cnt)	0.15–0.20 (cnt)	0.20–0.25 (cnt)	0.25–0.30 (cnt)	0.30–0.35 (cnt)	0.35–0.40 (cnt)	0.40–0.45 (cnt)	0.45–0.50 (cnt)	0.50–0.55 (cnt)	0.55–0.60 (cnt)	0.60–0.65 (cnt)	0.65–0.70 (cnt)	0.70–0.80 (cnt)	0.80–0.90 (cnt)
3734.0	4.353	0	0	0	0	0	0	0	0	0	0	0	0	0	0	0
3735.0	2.758	0	0	0	0	0	0	0	0	0	0	0	0	0	0	0
3736.0	6.917	0	0	0	0	0	0	0	0	0	0	0	0	0	0	0
3737.0	4.350	0	0	0	0	0	0	0	0	0	0	0	0	0	0	0
3738.0	4.476	0	0	0	0	0	0	0	0	0	0	0	0	0	0	0
3739.0	2.748	0	0	0	0	0	0	0	0	0	0	0	0	0	0	0
3740.0	7.067	0	0	0	0	0	0	1	0	0	0	0	0	0	0	0
3741.0	1.750	0	0	0	0	0	0	0	0	0	0	0	0	0	0	0
3742.0	3.625	0	0	0	0	0	0	0	0	0	0	0	0	0	0	0
3743.0	2.288	0	0	0	0	0	0	0	0	0	0	0	0	0	0	0
3744.0	3.010	0	0	0	0	0	0	0	0	0	0	0	0	0	0	0
3745.0	4.858	0	0	0	0	0	0	0	0	0	0	0	0	0	0	0
3746.0	5.630	0	0	0	0	0	0	0	0	0	0	0	0	0	0	0

Supplementary Methods

Microtektite counting in core MD90-0961

Core MD90-0961 (5°03.71' N, 73°52.57' E) was collected during the SEYMAMA research cruise of the R/V Marion Dufresne in 1990. The 45-m long core was retrieved on the eastern margin of the Chagos-Maldive-Laccadive Ridge, which extends over ~3000 km along the 73° meridian, at a water depth of 2450 m. The core is composed of calcareous nannofossil ooze with abundant foraminifera.

Typical microtektite concentrations amount to few counts per sample (~3 g). Therefore, standard sampling procedures based on few samples, which yield tektite profiles affected by large statistical noise (e.g., Glass and Koeberl, 2006), do not support the investigation of size segregation effects. Therefore, a particular effort has been undertaken to obtain sufficient counting statistics in three counting campaigns. For each sample, ~3 g of dry sediment was sieved at 120 mesh (125 μm) and subsequently treated with hydrochloric acid to remove carbonates. Microtektites where identified in the treated material and photographed under the optical microscope. Microtektite images were used to estimate individual sizes, which ranged between ~0.05 and ~0.9 mm. Counting results from the three campaigns, grouped into 15 size classes, are summarized in Tables S1–S3. Finally, a composite profile has been created by grouping the original data into 3 size classes with similar total counts: 0.05–0.2 mm, 0.2–0.45 mm, and 0.45–0.9 mm (Table S4).

The size distribution g_t of microtektites has been estimated by fitting total counts in the original size classes of Tables S1–S3 with a lognormal distribution (Fig. A1). The properties of the fitted g_t outside the 0.05–0.9 mm size range of counted microtektites are irrelevant, since g_t is used only within this range.

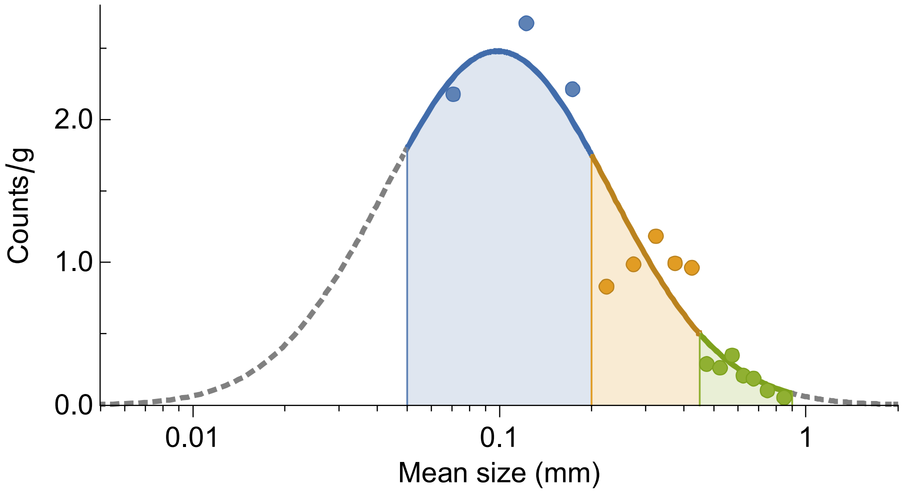

Figure A1. Total microtektite counts (dots) for the 15 size classes used in the original counting campaigns (Tables S1-S3), and least-squares fit with a lognormal size distribution (line). Shaded

areas represent the total contributions to the 0.05–0.2 mm, 0.2–0.45, and 0.49–0.9 mm size classes used for modeling.

The general diffusion-advection model

Consider a stationary sedimentary column characterized by a constant depth-dependent volume fraction ϕ_s, defined as the fraction of total volume occupied by solids. Mixing of solids in the upper surface mixed layer (SML) is characterized by the self-diffusion coefficient $D_s(z)$, where z is the depth below the sediment-water interface, and by the advection velocity $v_s = v_b + v_a$, which consists of two components: the burial velocity v_b due to continuous sedimentation, and the advection velocity v_a associated with non-local transport mechanisms in the SML. The associated downward flux of solids is given by

$$J_s = (v_b + v_a)\phi_s - D_s \frac{\partial \phi_s}{\partial z} \tag{1}$$

with boundary condition $J_s(0) = \rho_s^{-1} F_s$ at the sediment-water interface, where ρ_s is the density of sediment particles and F_s the incoming flux of sediment, including the material resuspended by bioturbation, as mass per unit of surface and time. Below the maximum bioturbation depth L, $D_s = 0$ and $v_a = 0$, so that $J_s = v_b \phi_s$ coincides with the burial flux. Because of mass conservation, J_s does not depend on depth, so that $v_b = J_s/\phi_s$ for $z > L$. If this definition of v_b is extended to the SML and substituted into eq. (1), the governing law

$$\phi_s v_a = D_s \frac{\partial \phi_s}{\partial z} \tag{2}$$

is obtained for the advective flux associated with the bulk transfer of sediment particles from more porous to less porous layers. This flux is also known as 'interphase mixing', as opposed to the 'intraphase mixing', which equalizes compositional differences.

Consider now a conservative solid tracer with volume fraction $\phi_t \ll \phi_s$. Its volume and mass-normalized concentrations are given by $C_v = \phi_t/\phi_s$ and $C = \rho_t \phi_t / \rho_s \phi_s$, respectively, with ρ_s being the density of sediment particles and ρ_t that of the tracer particles. The tracer flux in sediment is also governed by eq. (1); however, for convenience, an additional advective velocity v_t is introduced to describe the relative motion of tracer particles with respect to the bulk sediment, e.g., because of size segregation or selective ingestion by benthic organisms. In this case, the tracer flux gradient yields the diffusion-advection equation

$$\frac{\partial \phi_t}{\partial t} = \frac{\partial}{\partial z}\left[D_t \frac{\partial \phi_t}{\partial z} - (v_b + v_a - v_t)\phi_t\right], \tag{3}$$

in which D_t is the tracer diffusivity. If v_a is given by eq. (2), v_b by $J_s = v_b \phi_s$, and $C = \phi_t/\phi_s$, eq. (3) yields the general diffusion-advection equation

$$\phi_s \frac{\partial C}{\partial t} = \frac{\partial}{\partial z}\left[\phi_s D_t \frac{\partial C}{\partial z} + (D_t - D_s)\frac{\partial \phi_s}{\partial z} C - (J_s - \phi_s v_t)C\right]. \tag{4}$$

In the case of a tracer undergoing the same mixing processes as the bulk sediment, eq. (4) reduces to the classic diffusion-advection equation

$$\phi_\mathrm{s}\frac{\partial C}{\partial t}=\frac{\partial}{\partial z}\left[\phi_\mathrm{s}D_\mathrm{t}\frac{\partial C}{\partial z}-J_\mathrm{s}C\right]. \tag{5}$$

The tracer mass flux F at the sediment-water interface yields the boundary condition

$$F(t)=\rho_\mathrm{s}\left[(J_\mathrm{s}+\phi_\mathrm{s}v_\mathrm{t})C+(D_\mathrm{s}-D_\mathrm{t})\frac{\partial\phi_\mathrm{s}}{\partial z}C-\phi_\mathrm{s}D_\mathrm{t}\frac{\partial C}{\partial z}\right]_{z=0}. \tag{6}$$

Concentration profiles

Tracer concentration profiles below the SML are determined by the time evolution of the tracer concentration at $z=L$, which is governed by the diffusion-advection equation. The time-depth relation is described by the time

$$t_\mathrm{b}(z)=\int_L^z\frac{\mathrm{d}u}{v_\mathrm{b}(u)} \tag{7}$$

required for burial from the bottom of the SML to a given depth z. The inverse function ζ of t_b describes the depth $z=\zeta(t)$ reached by a tracer particle after a time t from its first appearance below SML. Given the evolution $C(L,t)$ of the tracer's mass concentration at $z=L$ resulting from an incoming flux $F(t)$, the concentration profile at a given time T is given by

$$C_z(z;T)=C(L,T-t_\mathrm{b}(z)). \tag{8}$$

In case of a tracer input event that is limited in time, and when T is much larger than the event duration, t_b can be replaced by the Taylor approximation

$$t_\mathrm{b}(z)=\int_L^{z_0}\frac{\mathrm{d}u}{v_\mathrm{b}(u)}+\int_{z_0}^{z}\frac{\mathrm{d}u}{v_\mathrm{b}(z_0+u)}\approx t_\mathrm{b}(z_0)+\frac{z-z_0}{v_\mathrm{b}(z_0)} \tag{9}$$

of eq. (7), with $z_0=\zeta(T)$ being the depth where the tracer is expected to appear. Then,

$$C_z(z;T)\approx C\left(L,\frac{\zeta(T)-z}{v_\mathrm{b}(\zeta(T))}\right). \tag{10}$$

Impulse response and age distribution

An impulsive tracer input at the time $t=-T$ before present is described by the incoming mass flux $F(t)=\Phi\delta(t-T)$, where Φ is the tracer fluence, that is, the total mass deposited per unit area. In the limit case of a purely advective SML $(D_\mathrm{s}=D_\mathrm{t}=0)$ with no size segregation $(v_\mathrm{t}=0)$, the tracer pulse will reach a given depth $z>L$ after a time $t=T_L+t_\mathrm{b}(z)$, where

$$T_L=\int_0^L\frac{\mathrm{d}u}{v_\mathrm{b}(u)+v_\mathrm{a}(u)} \tag{11}$$

is the advective escape time from the SML. Diffusive mixing in the SML causes a smearing of

the input impulse, so that the real escape time t_L is a random variable with different realizations for individual tracer particles. In this case, T_L is the deterministic component of the escape time. The time evolution of the tracer concentration at the bottom of the SML is described by the impulse response function

$$\mathfrak{I}(t) = C(L,t) \tag{12}$$

obtained from the solution $C(z,t)$ of the diffusion-advection equation with boundary condition $F(t) = \delta(t)$. A purely advective SML yields $\mathfrak{I}(t) = \delta(t - T_L)$, while in all other cases $\mathfrak{I}(t)$ is a function with domain $t \geq 0$ (because the time argument of C in eq. (12) must be positive) and unit integral over this domain (because of mass conservation).

In the framework of a microscopic description of tracer diffusion, individual tracer particles perform a biased 1D random walk with reflecting boundary at $z = 0$ (tracer particles cannot leave the sediment). In this case, $\mathfrak{I}$ is the probability density function that describes the first-passage time t_L of the particle across $z = L$. Once the particle has reached the bottom of the SML, it becomes definitively trapped in a sediment layer that is buried according to the age model described above. Arguments of $\mathfrak{I}$ smaller than the time T_L required to cross the SML by advection represent the finite probability of diffusion being faster than advection. The limit case of $t_L = 0$ corresponds to a particle that is instantaneously transported across the SML.

Using the above definition of $\mathfrak{I}$ and eq. (10), one obtains the concentration profile

$$C_z(z;T) \approx \Phi \mathfrak{I}\left(\frac{z_0 - z}{v_\mathrm{b}(z_0)}\right) \tag{13}$$

generated by an impulsive input of age T and fluence Φ, where $z_0 = \zeta(T)$ is the maximum depth reached by the tracer.

Solutions of the diffusion-advection equation for the homogeneous SML

Sediment mixing in a homogeneous SML of thickness L is described by a special case of eq. (4) with constant ϕ_s and D_t, which yields

$$\frac{\partial C}{\partial t} = D_\mathrm{t} \frac{\partial^2 C}{\partial z^2} - (v_\mathrm{b} - v_\mathrm{t}) \frac{\partial C}{\partial z}, \tag{14}$$

with boundary condition

$$F(t) = \rho_\mathrm{s} \phi_\mathrm{s} \left[(v_\mathrm{b} - v_\mathrm{t}) C - D_\mathrm{t} \frac{\partial C}{\partial z} \right]_{z=0}. \tag{15}$$

A special case of eq. (14–15) with $v_\mathrm{t} = 0$ was solved by Guinasso and Schink (1975). The tracer velocity v_t relative to the sediment is not assimilable with the burial velocity, since v_b is continuous across the lower boundary of the SML, while v_t, like D_t, is constant within the SML and zero below. The discontinuity of v_t at $z = L$ requires a different solution approach. If

solutions are limited to the $[0,L]$ interval where diffusion and selective advection of tracer particles occur, a boundary condition of the form

$$0=\left[v_\mathrm{t}C+D_\mathrm{t}\frac{\partial C}{\partial z}\right]_{z=L} \tag{16}$$

needs to be added at the bottom of the SML. This condition ensures that the tracer flux is continuous across $z=L$.

In the case of a tracer behaving as regular sediment particles ($D_\mathrm{t}=D_\mathrm{s}$, $v_\mathrm{t}=0$), the variable transformations $x=z/L$ and $t^*=v_\mathrm{b}t/L$ reduce eq. (14) to a nondimensionalized diffusion-advection equation of the form

$$\frac{\partial C}{\partial t^*}=G_\mathrm{s}\frac{\partial^2 C}{\partial x^2}-\frac{\partial C}{\partial x}, \tag{17}$$

which depends on a single parameter $G_\mathrm{s}=\mathrm{Pe}^{-1}=D_\mathrm{s}/Lv_\mathrm{b}$ corresponding to the inverse Péclet number (Guinasso and Schink, 1975). The Péclet number Pe is defined as the ratio between advective and diffusive transport over the typical length scale L of the mixed layer. A similar nondimensionalization is obtained for $v_\mathrm{t}>0$ upon replacing v_b in the definition of G_s with the net velocity $|v_\mathrm{b}-v_\mathrm{t}|$ of tracer particles with respect to $z=0$. In this case, the variable substitutions $x=z/L$ and $t^*=|v_\mathrm{b}-v_\mathrm{t}|t/L$ for $v_\mathrm{t}\neq v_\mathrm{b}$ give

$$\frac{\partial C}{\partial t^*}=G\frac{\partial^2 C}{\partial x^2}\mp\frac{\partial C}{\partial x}, \tag{18}$$

with $G=D_\mathrm{t}/L|v_\mathrm{b}-v_\mathrm{t}|$. The negative sign of $\partial C/\partial x$ in eq. (18) applies to the case of net downward ($v_\mathrm{t}<v_\mathrm{b}$) advection, while the positive sign applies to the upward ($v_\mathrm{t}>v_\mathrm{b}$) advection case. The boundary conditions at the sediment-water interface and at the bottom of the mixed layer are given by

$$\left[\pm C-G\frac{\partial C}{\partial x}\right]_{x=0}=\frac{F(t)}{\phi_\mathrm{s}\rho_\mathrm{s}|v_\mathrm{b}-v_\mathrm{t}|} \tag{19}$$

and

$$\left[wC+G\frac{\partial C}{\partial x}\right]_{x=1}=0, \tag{20}$$

respectively, with $w=|v_\mathrm{b}/v_\mathrm{t}-1|^{-1}$ being the normalized amplitude of the burial velocity discontinuity across $x=1$ caused by v_t.

The $v_\mathrm{t}=v_\mathrm{b}$ case must be treated separately, since $t^*=0$ is not a valid variable substitution. In this case, the variable transformations $x=z/L$ and $t^*=v_\mathrm{b}t/L$ yield the diffusion equation

$$\frac{\partial C}{\partial t^*}=G\frac{\partial^2 C}{\partial x^2} \tag{21}$$

with $G=D_\mathrm{t}/Lv_\mathrm{b}$ and boundary conditions

$$-G\frac{\partial C}{\partial x}\bigg|_{x=0}=\frac{F(t)}{\phi_s\rho_s v_b}, \qquad G\frac{\partial C}{\partial x}\bigg|_{x=1}=0. \tag{22}$$

Solution for an impulsive input flux

The calculation of the impulse response produced by eq. (18–22) begins by replacing the tracer flux pulse with the initial condition $C(x,0)=L^{-1}\delta(x)$, where the factor L^{-1} arises from the variable substitution for depth[1]. This initial condition represents the tracer concentration immediately after the tracer pulse has formed in an infinitely thin layer at the top of the sedimentary column. From this time on, $F(t)=0$ in eq. (20) and eq. (22). Let $\mathcal{G}(x,t^*)$ be the solution of the nondimensionalized diffusion-advection equation with $\mathcal{G}(x,0)=\delta(x)$, as in Guinasso and Schink (1975). If $v_t<v_b$, the substitution

$$\mathcal{G}(x,t^*)=\exp\left(\frac{2x-t^*}{4G}\right)c(x,t^*) \tag{23}$$

transforms eq. (18) into the diffusion equation

$$\frac{\partial c}{\partial t^*}=G\frac{\partial^2 c}{\partial x^2}, \tag{24}$$

with initial and boundary conditions

$$\begin{aligned} &c=\delta && ,\ t^*=0 \\ &\frac{\partial c}{\partial x}=\frac{1}{2G}c && ,\ x=0\ . \\ &\frac{\partial c}{\partial x}=-\frac{1+2w}{2G}c && ,\ x=1 \end{aligned} \tag{25}$$

A general solution of eq. (24) is obtained with the method of separation of variables assuming $c=X(x)T(t^*)$, which gives

$$\frac{T'}{GT}=\frac{X''}{X}. \tag{26}$$

Eq. (26) holds for all x and t if the left- and right-hand sides are equal to a constant. Calling this constant $-\lambda^2$, one obtains the ordinary differential equations

$$\begin{aligned} &T'+\lambda^2 GT=0 \\ &X''+\lambda^2 X=0 \end{aligned} \tag{27}$$

[1] The initial condition is expressed as $C(z,0)=\delta(z)$ in original coordinates, with the definition of the Dirac impulse as the limit $z_0\to 0$ of $z_0^{-1}f(z/z_0)$, where f is a regular function with unit integral. The variable substitution $x=z/L$ transforms the initial condition into $\mathcal{G}(x,0)=z_0^{-1}f(Lx/z_0)$ with the limit $\mathcal{G}(x,0)=L^{-1}\delta(x)$ for $z_0\to 0$.

with general solution

$$
\begin{aligned}
T(t^*) &= S e^{-G\lambda^2 t^*} \\
X(x) &= A\cos\lambda x + B\sin\lambda x
\end{aligned}
\tag{28}
$$

for $t^* \geq 0$ and $0 \leq x \leq 1$, where S, A, and B are arbitrary constants. The spatial boundary conditions in eq. (25) impose $B\lambda = A/2G$ and

$$
\cot\lambda = \frac{1}{1+w}\left[G\lambda - \frac{1+2w}{4G\lambda}\right].
\tag{29}
$$

Eq. (29) has one real solution λ_k every continuous interval $](k-1)\pi, k\pi[$ of $\cot\lambda$ with $k=$ $k=1,2,\ldots$, which must be found numerically. These are the eigenvalues to the eigenfunctions $X_k T_k$ with $X_k = \cos\lambda_k x + \sin\lambda_k x/2G\lambda_k$ and $T_k = \exp(-G\lambda_k^2 t^*)$. The general solution of the diffusion equation is a linear combination of these eigenfunctions, that is

$$
c(x,t^*) = \sum_{k=1}^{\infty} a_k X_k(x) e^{-G\lambda_k^2 t^*} .
\tag{30}
$$

The coefficients a_k must satisfy the initial condition

$$
c(x,0) = \sum_{k=1}^{\infty} a_k X_k(x) = \delta(x)
\tag{31}
$$

at $t^* = 0$ for $0 \leq x \leq 1$. The eigenfunctions fulfill the orthogonality relations

$$
\int_0^1 X_k(x) X_l(x)\,\mathrm{d}x = \delta_{kl}\gamma_k
\tag{32}
$$

with $\delta_{kl} = 1$ for $k = l$ and 0 else, and

$$
\gamma_k = \frac{1}{2} + \frac{1}{8G^2\lambda_k^2} + \frac{1+w}{2G\lambda_k^2} + \frac{w}{4G^2\lambda_k^3}\frac{\cot\lambda_k - 2G\lambda_k}{1+\cot^2\lambda_k}.
\tag{33}
$$

Multiplication of eq. (31) with X_l, integration over $0 \leq x \leq 1$, and use of the selection property of the Dirac impulse yields $a_l = \gamma_l^{-1}$. The solution of the diffusion-advection equation for $v_\mathrm{t} < v_\mathrm{b}$ is thus finally given by

$$
\mathcal{G}(x,t^*) = \sum_{k=1}^{\infty} \gamma_k^{-1}\left[\cos\lambda_k x + \frac{1}{2G\lambda_k}\sin\lambda_k x\right] e^{x/2G - (1/4G + G\lambda_k^2)t^*} .
\tag{34}
$$

In the limit case of $G \to \infty$, one has $\lambda_k = k\pi$, $\gamma_1 = 1/2$, and $\gamma_{k>1} = 0$, so that $\mathcal{G}(x,t^*) =$ $2^{-1} e^{(2x - t^*)/4G}$ is a pure exponential function.

The $v_\mathrm{t} > v_\mathrm{b}$ case can be solved in a similar manner, using the substitution

$$
\mathcal{G}(x,t^*) = \exp\left(\frac{-2x - t^*}{4G}\right) c(x,t^*)
\tag{35}
$$

with temporal and spatial boundary conditions

$$
\begin{aligned}
&c = \delta &&, t^* = 0 \\
&\frac{\partial c}{\partial x} = -\frac{1}{2G}c &&, x = 0 \ . \\
&\frac{\partial c}{\partial x} = \frac{1-2w}{2G}c &&, x = 1
\end{aligned} \tag{36}
$$

Application of the spatial boundary conditions on $c = X(x)T(t^*)$ with X and T defined in eq. (28) yields $B\lambda = -A/2G$ and

$$
\cot\lambda = \frac{1}{w-1}\left[G\lambda + \frac{2w-1}{4G\lambda}\right]. \tag{37}
$$

Eq. (37) defines one eigenvalue λ_k for every continuous interval $](k-1)\pi, k\pi[$ of $\cot\lambda$ with $k = 1,2,\ldots$, which must be found numerically. The eigenvalues are always real for $k > 1$. The first eigenvalue is real if $G \geq 1/2$ and $w \geq (4G-1)/(4G-2)$, and imaginary otherwise. The imaginary eigenvalue $\lambda = i\lambda_1$ defines the eigenfunction X_1T_1 with

$$
\begin{aligned}
&T_1 = S e^{+G\lambda_1^2 t^*} \\
&X_1 = A\cosh\lambda_1 x + iB\sinh\lambda_1 x
\end{aligned}, \tag{38}
$$

which satisfies the spatial boundary conditions in eq. (36) if $iB\lambda_1 = -A/2G$ and

$$
\coth\lambda_1 = \frac{1}{w-1}\left[\frac{2w-1}{4G\lambda_1} - G\lambda_1\right]. \tag{39}
$$

Using the same approach for the calculation of the integrals of X_k^2 as for $v_\mathrm{t} < v_\mathrm{b}$, the solution of the diffusion-advection equation for $v_\mathrm{t} > v_\mathrm{b}$ becomes

$$
\mathcal{G}(x,t^*) = \sum_{k=1}^{\infty} \gamma_k^{-1}\left[\cos\lambda_k x - \frac{1}{2G\lambda_k}\sin\lambda_k x\right] e^{-x/2G-(1/4G+G\lambda_k^2)t^*} \tag{40}
$$

with

$$
\gamma_k = \frac{1}{2} + \frac{1}{8G^2\lambda_k^2} + \frac{w-1}{2G\lambda_k^2} - \frac{w}{4G^2\lambda_k^3}\frac{\cot\lambda_k + 2G\lambda}{1+\cot^2\lambda_k} \tag{41}
$$

if $G \geq 1/2$ and $w \geq (4G-1)/(4G-2)$, or

$$
\begin{aligned}
\mathcal{G}(x,t^*) = {}& \gamma_1^{-1}\left[\cosh\lambda_1 x - \frac{1}{2G\lambda_k}\sinh\lambda_1 x\right] e^{-x/2G-(1/4G-G\lambda_1^2)t^*} + \\
& \sum_{k=2}^{\infty} \gamma_k^{-1}\left[\cos\lambda_k x - \frac{1}{2G\lambda_k}\sin\lambda_k x\right] e^{-x/2G-(1/4G+G\lambda_k^2)t^*}
\end{aligned} \tag{42}
$$

with

$$
\gamma_1 = \frac{1}{2} - \frac{1}{8G^2\lambda_1^2} - \frac{w-1}{2G\lambda_1^2} + \frac{w}{4G^2\lambda_1^3}\frac{\coth\lambda_1 - 2G\lambda_1}{\coth^2\lambda_1 - 1} \tag{43}
$$

in the other cases.

The last case to discuss is that of $v_\mathrm{t} = v_\mathrm{b}$, which describes a purely diffusive process. The definition of a unitless depth $x = z/L$ and time $t^* = v_\mathrm{b} t/L$ yields

$$\frac{\partial \mathcal{G}}{\partial t^*} = G \frac{\partial^2 \mathcal{G}}{\partial x^2}, \tag{44}$$

with $G = D_\mathrm{t}/L v_\mathrm{b}$, subjected to the initial and boundary conditions

$$\begin{aligned} &\mathcal{G} = \delta && , \ t^* = 0 \\ &\frac{\partial \mathcal{G}}{\partial x} = 0 && , \ x = 0 \ . \\ &\frac{\partial \mathcal{G}}{\partial x} = -\frac{1}{G}\mathcal{G} && , \ x = 1 \end{aligned} \tag{45}$$

Application of the spatial boundary conditions on $\mathcal{G} = X(x)T(t^*)$ with X and T defined in eq. (28) yields $B = 0$ and real eigenvalues λ_k satisfying $\cot\lambda_k = G\lambda_k$. The corresponding solution is

$$\mathcal{G}(x,t^*) = \sum_{k=1}^{\infty} \gamma_k^{-1}\, e^{-G\lambda_k^2 t^*} \cos\lambda_k x \tag{46}$$

with

$$\gamma_k = \frac{1}{2} + \frac{G}{2(1+G^2\lambda_k^2)}. \tag{47}$$

The impulse response

As mentioned in Section 1, the motion of individual particles is described by a Wiener process with constant drift starting at $(t,z) = (0,0)$ and ending at $(t,z) = (t_L, L)$, where t_L is the escape time. The boundary conditions impose a reflecting barrier at $z = 0$ and a partially absorbing, or elastic, barrier at $z = L$ (Dominé, 1996). The age T of tracer particles found at a depth $z \geq L$ below the mixed layer is related to t_L by $T = t_L + (z-L)/v_\mathrm{b}$. The probability density function of the nondimensionalized escape time $t_L^* = |v_\mathrm{b} - v_\mathrm{t}|t/L$ is determined by the survival probability

$$\mathcal{S}(t^*) = \frac{1}{L}\int_0^L \mathcal{G}(z/L, t^*)\,\mathrm{d}z = \int_0^1 \mathcal{G}(x,t^*)\,\mathrm{d}x, \tag{48}$$

defined as the probability that a tracer particle initially localized at $x = 0$ by $G(x,0) = \delta(x)$ remains in the SML for a time t^*. The probability that this particle escapes the mixed layer between t^* and $t^* + \mathrm{d}t^*$ is then given by $\mathcal{E}(t^*)\mathrm{d}t^* = \mathcal{S}(t^*) - \mathcal{S}(t^* + dt^*)$, where $\mathcal{E}$ is the probability density function of the nondimensionalized escape time t_L^*. Using eq. (48),

$$\mathcal{E}(t^*) = -\frac{\partial}{\partial t^*}\int_0^1 \mathcal{G}(x,t^*)\,\mathrm{d}x. \tag{49}$$

Furthermore, it can be verified that

$$\mathcal{G}(1,t^*)=\frac{|v_\mathrm{b}-v_\mathrm{t}|}{v_\mathrm{b}}\mathcal{E}(t^*), \tag{50}$$

as expected from the equivalency between the diffusion-advection equation and its microscopic description in terms of a biased random walk[2]. Application of eq. (49) to the solutions for $\mathcal{G}$ obtained above yields

$$\mathcal{E}(t^*)=e^{1/2G}\sum_{k=1}^{\infty}\frac{1+4G^2\lambda_k^2}{4\gamma_k G\lambda_k}e^{-(1/4G+G\lambda_k^2)t^*}\sin\lambda_k \tag{51}$$

for $v_\mathrm{t}<v_\mathrm{b}$,

$$\mathcal{E}(t^*)=e^{-1/2G}\sum_{k=1}^{\infty}\frac{1+4G^2\lambda_k^2}{4\gamma_k G\lambda_k}e^{-(1/4G+G\lambda_k^2)t^*}\sin\lambda_k \tag{52}$$

for $v_\mathrm{t}>v_\mathrm{b}$ (with $i\lambda_1$ instead of λ_1 if the first eigenvalue is imaginary), and

$$\mathcal{E}(t^*)=\sum_{k=1}^{\infty}\frac{G\lambda_k}{\gamma_k}e^{-G\lambda_k^2 t^*}\sin\lambda_k \tag{53}$$

for $v_\mathrm{t}=v_\mathrm{b}$, respectively. The simpler form taken by the eigenfunction expansion of $\mathcal{E}$ yields a more convenient formulation of $\mathcal{G}$, and therefore of the impulse response, given by

$$\mathfrak{I}(t)=\frac{\eta v_\mathrm{b}}{L}\mathcal{E}\left(\frac{\eta v_\mathrm{b}}{L}t\right), \tag{54}$$

with $\eta=1$ for $v_\mathrm{t}=v_\mathrm{b}$, and $\eta=|1-v_\mathrm{t}/v_\mathrm{b}|$ for $v_\mathrm{t}\neq v_\mathrm{b}$ (Fig. A2). The escape time distribution $\mathcal{E}$ approaches an exponential function with decay time determined by the first eigenvalue λ_1 if $G\to\infty$ (pure diffusion), and a Gaussian function with expectation $t^*=1$ and vanishing standard deviation if $G\to 0$ (pure advection). A certain similarity exists between $\mathcal{E}$ and the inverse Gaussian distribution

$$\mathfrak{G}(t)=\frac{L}{\sqrt{2\pi D t^3}}\exp\left(-\frac{(L-vt)^2}{2Dt}\right), \tag{55}$$

[2] Because of mass conservation, integration of the tracer concentration $C(z,t)$ generated by an impulsive flux $\Phi\delta(t)$ with fluence Φ must be equal to Φ. Below the mixed layer, depth and time are related by the burial velocity v_b, so that

$$\int_0^\infty C(L,t)\mathrm{d}t=\frac{L}{|v_\mathrm{b}-v_\mathrm{t}|}\int_0^\infty C(L,t^*)\mathrm{d}t^*=\frac{\Phi}{v_\mathrm{b}}.$$

Using $C(z,t)=L^{-1}\mathcal{G}(z/L,t^*)$, which follows from the definition of $\mathcal{G}$, the above equation becomes

$$\int_0^\infty \mathcal{G}(L,t^*)\mathrm{d}t^*=\frac{|v_\mathrm{b}-v_\mathrm{t}|}{v_\mathrm{b}}\Phi.$$

Given the equivalency between diffusion-advection and random walk models, $\Phi=1$ defines the total probability 1 of the escape time, and thus the integral of $\mathcal{E}(t^*)$, yielding eq. (50).

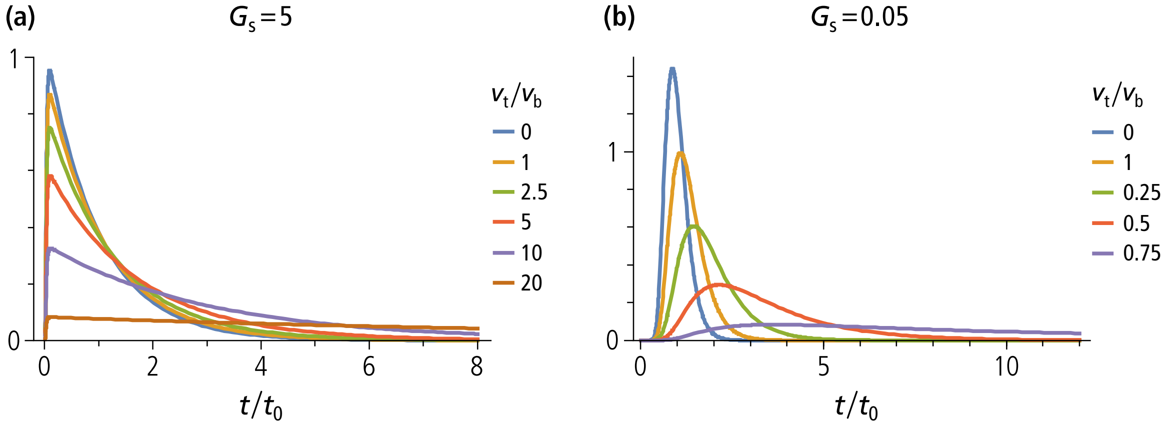

Figure A2. Impulse responses of a homogeneous SML for two values of the inverse Péclet number G_s (**a, b**), and selected ratios between the tracer segregation velocity v_t and the burial velocity v_b; $t_0 = L/v_b$ is the mean transit time of the bulk sediment.

which describes the first passage time through $z = L$ of a drifting Wiener process $Z_t = vt + \sigma W_t$ with no barriers, where v is the drift velocity, W_t the standard Brownian motion with variance $\sigma^2 t = Dt$, and D the equivalent diffusion coefficient. This similarity, however, is only apparent, since $\mathfrak{G}$ is defined only for $v \geq 0$ and its expectation diverges at $v = 0$, due to the lack of a reflecting barrier that keeps the diffusing particle at a finite distance from the passage threshold.

The eigenfunction expansion of $\mathcal{E}$ is an infinite series of terms with exponential time decay, which does not converge for $t^* = 0$ or $G = 0$. Rapid convergence, however, occurs for sufficiently large values of t^* and G. Moreover, $\mathcal{E}$ can be adequately approximated by truncating the series expansion to the first n terms at any $t^* > 0$ and $G > 0$ for a sufficiently large n. In this case, there is a threshold time $t_0^*(G,n) = \varepsilon(n) G^{-1}$, with $\varepsilon \sim n^{-1}$, below which $\mathcal{E}(t^*)$ becomes negative (Fig. A3a). This threshold represents a practical lower limit for the numerical support of $\mathcal{E}$ (Fig. A3b). For small values of G, numerical instabilities occur even for $t^* > t_0^*$, due to the small net result of adding terms with alternating sign and very large amplitude (Fig. A3c,d). In these cases, arbitrary numerical precision (e.g., using *Mathematica*) is required for a correct evaluation of $\mathcal{E}$.

The nondimensionalized escape time expectation is defined by

$$\langle t_L^* \rangle = \lim_{\varepsilon \to 0} \int_\varepsilon^\infty \mathcal{E}(t)\, t \, \mathrm{d}t \,, \tag{56}$$

where the limit avoids convergence problems at $t^* \to 0$. Integration of the exponential terms in eq. (51–53) gives

$$\langle t_L \rangle = \frac{L}{v_b - v_t} \lim_{\varepsilon \to 0} e^{1/2G} \sum_{k=1}^{\infty} \frac{\sin \lambda_k}{\gamma_k \lambda_k} \frac{4G + (1 + 4G^2 \lambda_k^2)\varepsilon}{1 + 4G^2 \lambda_k^2} e^{-(1/4G + G\lambda_k^2)\varepsilon} \tag{57}$$

for $v_t < v_b$,

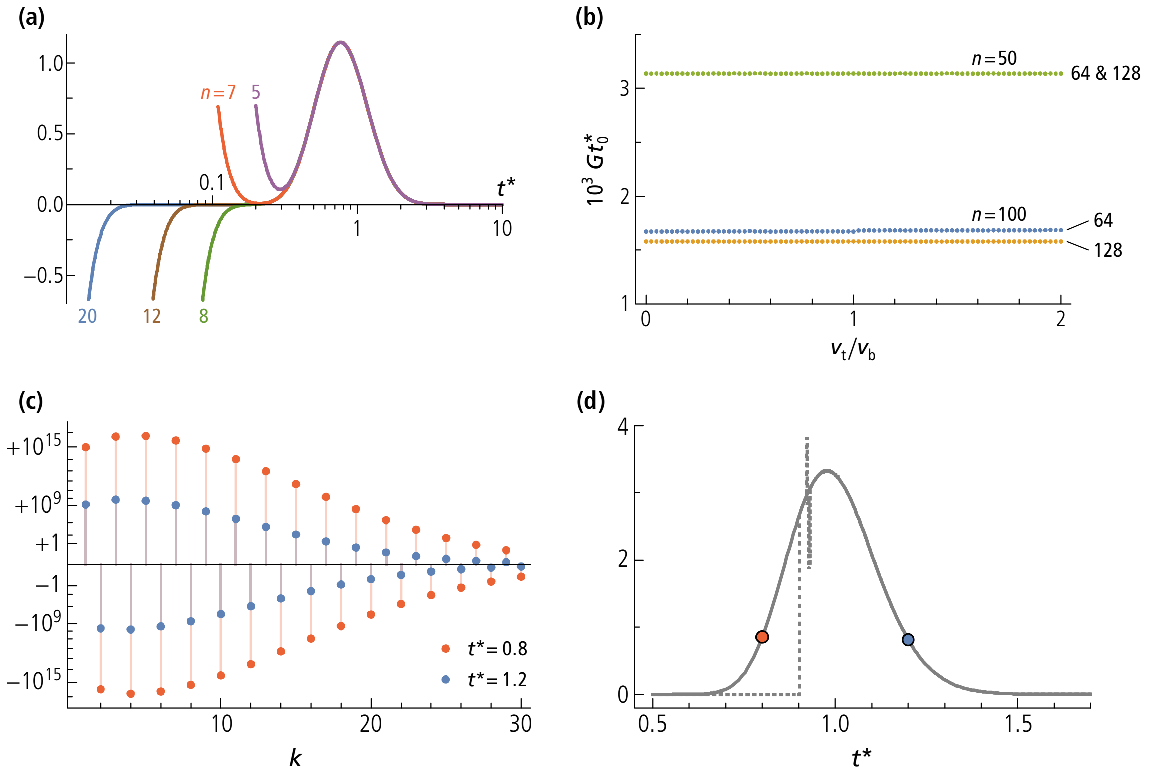

Figure A3. **(a)** Numerical evaluations of $\mathcal{E}(t^*)$ for $G=0.1$ and $v_\mathrm{t}=0$, using the first n terms of the eigenfunction expansion. Notice the change of sign occurring between $n=7$ and 8 for $t^*\to 0$. **(b)** Nondimensionalized time t_0^* below which eigenfunction expansions of $\mathcal{E}(t^*)$ truncated at $n=50$ and $n=100$ become negative, evaluated using 64 and 128 digit precision. Results obtained with a given digit precision depend only on n and G, regardless of v_t. **(c)** First 30 eigenfunctions of $\mathcal{E}(t^*)$ (the individual series terms in eq. 51) for $G_0=0.0075$ and $v_\mathrm{t}=0$, and two selected values of t^*. Notice the quasi-logarithmic scale used to represent alternating terms spanning 16 orders of magnitude. **(d)** Numerical evaluation of $\mathcal{E}(t^*)$ as sum of the eigenfunctions shown in (c), obtained in *Mathematica* using 16-digit precision (dashed line) and 32-digit precision (solid line).

$$\langle t_L\rangle=\frac{L}{v_\mathrm{t}-v_\mathrm{b}}\lim_{\varepsilon\to 0}e^{-1/2G}\sum_{k=1}^{\infty}\frac{\sin\lambda_k}{\gamma_k\lambda_k}\frac{4G+(1+4G^2\lambda_k^2)\varepsilon}{1+4G^2\lambda_k^2}e^{-(1/4G+G\lambda_k^2)\varepsilon} \tag{58}$$

for $v_\mathrm{t}>v_\mathrm{b}$ (with $i\lambda_1$ instead of λ_1 if the first eigenvalue is imaginary), and

$$\langle t_L\rangle=\frac{L}{v_\mathrm{b}}\lim_{\varepsilon\to 0}\sum_{k=1}^{\infty}\frac{\sin\lambda_k}{\gamma_k\lambda_k}\frac{1+G\lambda_k^2\varepsilon}{G\lambda_k^2}e^{-G\lambda_k^2\varepsilon} \tag{59}$$

for $v_\mathrm{t}=v_\mathrm{b}$. In absence of size segregation effects ($v_\mathrm{t}=0$), the expected escape time of tracer particles coincides with the transit time L/v_b through the mixed layer resulting from the burial velocity. Grain size segregation increases the escape time of large particles, reflecting their lessened burial velocity. Accordingly, $\langle t_L\rangle$ is a monotonic function of v_t, which increases at a double exponential rate once v_t exceeds a certain threshold determined by G_s (Fig. A4). In quasi-advective regimes ($G_\mathrm{s}\ll 1$), the increase of $\langle t_L\rangle$ is particularly rapid once v_t exceeds v_b, because the net upward velocity is hardly overcome by diffusion.

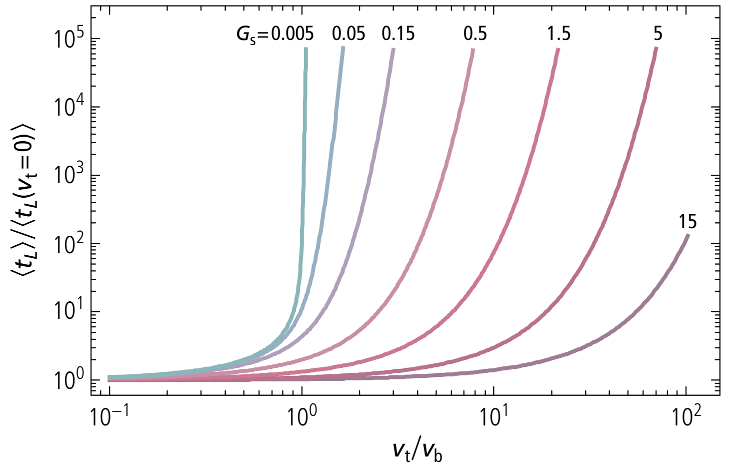

Figure A4. Expected escape time $\langle t_L \rangle$ of a tracer particle with segregation velocity v_t, normalized by the $v_t = 0$ case, as a function of v_t / v_b, for selected values of the inverse Péclet number G_s of the bulk sediment. These values correspond to a mixed layer with thickness $L = 10$ cm, burial velocity $v_b = 2$ cm/kyr, and bulk diffusion coefficients $D_s = 0.1$, 1, 3, 10, 30, 100, and 300 cm^2/kyr, respectively. Notice the logarithmic scale on the vertical axis.

Impulsive tracer profiles in the historical layer

Consider a tracer with impulsive input $F = \Phi\delta(t)$ at $t = 0$, where Φ is the fluence of this event, expressed as total deposited amount (e.g., particle counts or total mass) per unit of area. In a sediment with constant porosity, the resulting mass concentration below a SML with thickness L is given at any time $t > 0$ by

$$C_z(z,t) = \frac{\Phi}{\rho_s J_s} E\left(t - \frac{z-L}{v_b}\right) \tag{60}$$

where E is the probability density function of the escape time of tracer particles with given size from the mixed layer, $(z-L)/v_b$ the additional time required for a sediment particle to be buried from just below the mixed layer to $z > L$, and $J_s = \phi_s v_b$ the depth-independent sediment flux. The negative sign of z in eq. (60) means that the tail of E is directed upwards: tracer particles that required more time to escape the mixed layer entered the historical layer at a later time and are buried less deep. Using the nondimensionalized results derived above, eq. (60) becomes

$$C_z(z,t) = \frac{\Phi}{\rho_s J_s \tau} \mathcal{E}\left(\frac{v_b t - z + L}{v_b \tau}\right), \tag{61}$$

where $\mathcal{E}(t^*)$ is the probability density function of the nondimensionalized escape time t^*, given by eq. (51–53), and $\tau = \mathrm{d}t/\mathrm{d}t^*$ a time constant defined by the variable transformation $t \to t^*$, that is:

$$\tau = \begin{cases} L v_b^{-1} & , v_t = v_b \\ L|v_b - v_t|^{-1} & , \text{else} \end{cases}. \tag{62}$$

Sediment compaction below the SML causes a compression of C_z. This effect is accounted by replacing the burial time $(z-L)/v_\mathrm{b}$ below the SML with the corresponding expression $t_\mathrm{b}(z)$ derived from the age model in eq. (7). Then, eq. (61) becomes:

$$C_z(z,t)=\frac{\Phi}{\rho_\mathrm{s}J_\mathrm{s}\tau}\mathcal{E}\left(\frac{t-t_\mathrm{b}(z)}{\tau}\right). \tag{63}$$

At large depths compaction over the limited range of C_z is negligible, so that t_b is conveniently approximated by the Taylor expansion $t_\mathrm{b}(z)=t_\mathrm{b}(z_0)+(z-z_0)/v_\mathrm{b}$ about a reference depth z_0 within the range of C_z. In this case, eq. (63) becomes

$$C_z(z,t)=\frac{\Phi}{\rho_\mathrm{s}J_\mathrm{s}\tau}\mathcal{E}\left(\frac{v_\mathrm{b}(z_0)t-z}{v_\mathrm{b}(z_0)\tau}-\frac{v_\mathrm{b}(z_0)t_\mathrm{b}(z_0)-z_0}{v_\mathrm{b}(z_0)\tau}\right) \tag{64}$$

with $\tau=L/\eta v_\mathrm{b}(0)$ and $\eta=1$ for $v_\mathrm{t}=v_\mathrm{b}$, or $\eta=|1-v_\mathrm{t}/v_\mathrm{b}|$ for $v_\mathrm{t}\neq v_\mathrm{b}$. Introducing the compaction factor $\phi=\phi_\mathrm{s}(z_0)/\phi_\mathrm{s}(0)\geq 1$, defined as the ratio between sediment volume fraction at z_0 and within the SML, eq. (64) finally becomes

$$C_z(z,t)=\frac{\Phi}{\rho_\mathrm{s}\phi_\mathrm{s}}\frac{\eta\phi}{L}\mathcal{E}\left(\frac{\eta\phi}{L}(v_\mathrm{b}t-v_\mathrm{b}t_\mathrm{b}+z_0-z)\right) \tag{65}$$

with ϕ_s and v_b being evaluated at $z=z_0$.

Size segregation effects can be quantified by evaluating the difference $\langle\Delta z\rangle$ between the expected depths of concentration profiles corresponding to the actual tracer and to a tracer with $v_\mathrm{t}=0$, respectively. In this case,

$$\langle\Delta z\rangle=\langle t_L\rangle v_\mathrm{b}(z_0)-\frac{L}{\eta\phi} \tag{66}$$

where $\langle t_L\rangle$ is the expected escape time given by eq. (57–59) (Fig. A4). Increasing segregation effects move the concentration profiles upwards and makes them broader, similarly to what occurs in the time domain (Fig. A2). This effect grows rapidly when v_t increases beyond v_b, leading to homogeneous spreading of large tracer particles over the whole sedimentary column.

Steady-state solution of the diffusion-advection equation

Steady-state solutions of the diffusion-advection equation for a homogeneous SML are obtained by solving eq. (17–19) for a constant flux F of tracer particles. In this case, C does not depend on time, and the nondimensionalized diffusion-advection equation becomes

$$G\frac{\partial^2 C}{\partial x^2}\mp\frac{\partial C}{\partial x}=0 \tag{67}$$

with boundary conditions

$$\pm C - G\frac{\partial C}{\partial x} = \frac{F}{|v_\mathrm{b} - v_\mathrm{t}|}, \quad x = 0$$
$$wC + G\frac{\partial C}{\partial x} = 0, \quad x = 1 \quad . \tag{68}$$

The general solution of eq. (67) is

$$C = C_0 + A\left[\cosh(x/G) \pm \sinh(x/G)\right] \tag{69}$$

with the boundary conditions yielding

$$C_0 = \pm\frac{F}{|v_\mathrm{b} - v_\mathrm{t}|}$$
$$A = -\frac{F}{|v_\mathrm{b} - v_\mathrm{t}|}\frac{v_t}{v_\mathrm{b}}\frac{1}{\sinh(1/G) \pm \cosh(1/G)} \quad . \tag{70}$$

The corresponding tracer concentrations at the sediment-water interface and at the bottom of the mixed layer,

$$C(0) = \frac{\pm F}{|v_\mathrm{b} - v_\mathrm{t}|}\left[1 - \frac{v_t}{v_\mathrm{b}}\frac{1}{\cosh(1/G) \pm \sinh(1/G)}\right],$$
$$C(1) = \frac{F}{v_\mathrm{b}} \tag{71}$$

define the concentration ratio

$$R = \frac{C(0)}{C(1)} = \frac{1}{1 - v_\mathrm{t}/v_\mathrm{b}}\left[1 - \frac{v_t}{v_\mathrm{b}}\frac{1}{\cosh(1/G) \pm \sinh(1/G)}\right], \tag{72}$$

which is a measure for the concentration gradient generated by grain size segregation. This gradient is fully contained within the mixed layer, since tracer concentrations cannot change below the mixed layer. The case $v_\mathrm{t} = v_\mathrm{b}$ is singular and must be treated differently. Defining $G = D/Lv_\mathrm{b}$, as for the impulse response model, one obtains

$$R = 1 + \frac{1}{G}. \tag{73}$$

The concentration gradient increases rapidly with increasing segregation, similarly to the escape time (Fig. A5). This can be qualitatively explained by mass conservation: in the stationary case, the tracer flux is constant and equal at all depths, so that the lower sinking rate of large tracer particles inside the mixed layer must be compensated by a steeper concentration gradient. This leads to the selective accumulation of larger particles near the sediment-water interface. A notable example of this phenomenon is the accumulation of ferromanganese nodules on the ocean floor, with a steep concentration gradient in the top ~40 cm of the sedimentary column (Finney et al., 1984).

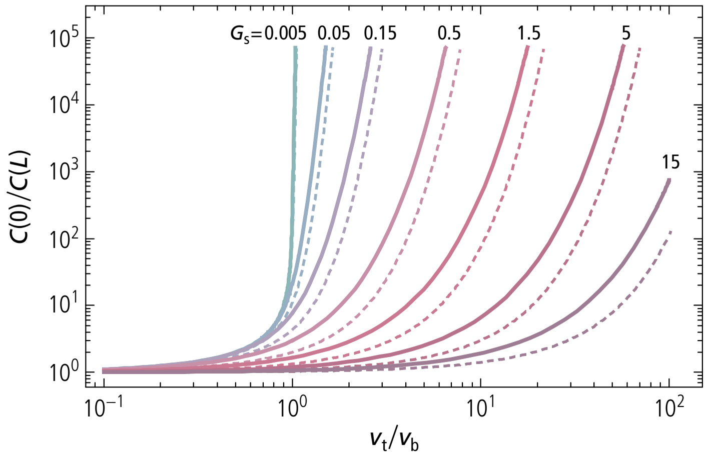

Figure A5. Ratio between the tracer concentration at the sediment-water interface and at the bottom of the mixed layer, as a function of v_t/v_b, for selected values of the inverse Péclet number G_s (solid lines). The normalized escape time expectation of Fig. S3 is shown for comparison (dashed lines). The values of G_s correspond to a mixed layer with thickness $L=10$ cm, burial velocity $v_b=2$ cm/kyr, and bulk diffusion coefficients $D_s=0.1$, 1, 3, 10, 30, 100, and 300 cm^2/kyr, respectively. Notice the logarithmic scale on the vertical axis.

Size segregation model

Several studies report the tendency of larger particles initially deposited on the surface of bioturbated sediment to penetrate less rapidly into the sediment (e.g., Thomson et al., 1995). This effect has been attributed to the preferential ingestion of finer particles by deposit feeders (Shull and Yasuda, 2001), which, in a diffusive bioturbation model, leads to a grain size-dependent diffusion coefficient. Experiments with sorted glass beads suggest a power law dependency of the form $D_t \propto s^{-q}$, where s is the bead size and $q \approx 0.52$ (Wheatcroft et al., 1992). The much stronger ingestion selectivity of deposit feeders ($q \approx 2.8$ according to data from Shull and Yasuda, 2001) suggests that less selective mixing mechanisms must be responsible for the observed size dependence of diffusion.

The diffusion of particles percolating through random media, of which sediments are an example, is controlled by microstructures, which are often fractal over a finite range of length scales. This gives raise to anomalous diffusion, which depends on the ratio between particle size and the length scale of pores. For instance, the diffusion coefficient in fractal soil structures has been modeled with $D = D_0 (r/r_0)^{-q}$, where r is a length scale, D_0 the effective Fickian diffusion coefficient at scales smaller than the smallest size r_0 of fractal structures, and q an exponent comprised between ~0.28 and ~0.66 (Anderson et al., 2000). The glass bead experiments of Wheatcroft et al. (1992) lie in this range. For comparison, the diffusion coefficient of Brownian diffusion is characterized by $q=1$, while $q=0$ is expected for infinite fractals.

Because a power law dependence of the diffusivity on grain size is obtained from both biogenic and abiogenic mixing mechanisms, the tracer diffusion coefficient is modeled assuming

$$D_\text{t} = \alpha D_\text{s} \tag{74}$$

with $\alpha = \psi^{-q}$ and $0 < q < 1$, where D_s is the solid self-diffusion coefficient of the bulk sediment, and $\psi = s/s_0$ the ratio between the size s of tracer particles, and the characteristic (mean or median) size s_0 of sediment particles.

The advective component of size segregation is proportional to the segregation velocity v_t (positive upwards) of large tracer particles with respect to the bulk sediment. Advective segregation, or granular convection, can be explained by the selective downward percolation of smaller grains through pores when particle assemblages are agitated or mixed. The resulting tendency of large particles to rise to the top is also known as Brazil nut phenomenon. The most prominent example in sedimentary environments is the persistence of ferromanganese nodules at the sediment surface (Piper and Flower, 1980). Granular convection is also affected by density, with the counterintuitive tendency of large, less dense particles to raise less rapidly. In analogy with laboratory experiments (Kudrolli, 2004), the grain size dependence of v_t is modelled by by the ramp function

$$v_\text{t} \propto \begin{cases} 0 & , \psi \le \psi_\text{c} \\ \psi - \psi_\text{c} & , \psi > \psi_\text{c} \end{cases} \tag{75}$$

where ψ_c is threshold value of the size ratio above which size separation occurs. Very different experimental settings yield similar values of ψ_c around 3 (Kudrolli, 2004). In dry bed vibrating experiments, the relative upward velocity of a large sphere is proportional to the vibration frequency in the low-frequency limit[3]. A similar behavior is obtained also for cohesive materials simulated by wet powders, whereby increasing cohesion forces are found to decrease the segregation effect. If episodic sediment disturbances produce, on average, a certain size segregation effect, it can be assumed that these effects add in time, in analogy with the microscopic particle displacements underlying diffusive models of bioturbation-driven sediment mixing. In this case, v_t is proportional to the frequency of bioturbation events and thus to D_s, regardless of micromechanical details, and eq. (75) can be rewritten as

$$v_\text{t} = \beta(\psi) D_\text{s} \tag{76}$$

with

$$\beta(\psi) = \begin{cases} 0 & , \psi \le \psi_\text{c} \\ \beta_0 (\psi - \psi_\text{c}) & , \psi > \psi_\text{c} \end{cases}, \tag{77}$$

where β_0 is a constant coefficient with the unit of an inverse length.

[3] Vanel et al. (1997) found that the rise time T of a large sphere in a granular bed is proportional to $[\exp(\nu^2/\nu_\text{c}^2) - 1](\Gamma - \Gamma_\text{c})^{-\beta} \nu^{-1}$, where ν is the vibrating frequency, ν_c a critical value of ν, Γ the unitless acceleration of vibrations, Γ_c a critical value of Γ, and $\beta \approx 2$. The limit of this expression for $\nu \to 0$ is $T \propto \nu^{-1}$, so that the rising velocity is proportional to ν.

Effect of size segregation on age models

Consider tracer particles whose age coincides with that of deposition, up to a constant offset. In the case of an impulsive deposition event, particles with identical age will be spread over a range of depths upon burial, due to the statistical nature of the time required to escape the SML. Using eq. (54) and eq. (63–65), the probability of finding a particle of deposition age T at a depth $z \geq L$ is given by

$$\mathcal{A}(T) = \frac{\phi\eta v_\mathrm{b}}{L}\,\mathcal{E}\left(\frac{\phi\eta v_\mathrm{b}}{L}(T - t_\mathrm{b}(z))\right), \tag{78}$$

with v_b being the burial velocity at z and t_b the burial time defined by eq. (7). The expected age associated with eq. (78) is

$$\langle T\rangle = t_\mathrm{b}(z) + \frac{L}{\phi\eta v_\mathrm{b}}\langle t^*\rangle, \tag{79}$$

where the expectation $\langle t^*\rangle$ of $\mathcal{E}$ is a function of $v_\mathrm{t}/v_\mathrm{b}$ and thus of tracer particle size (Fig. A4). Tracer particles whose size does not exceed the segregation threshold behave like the bulk sediment, which means that $v_\mathrm{t} = 0$, $\eta = 1$, and $\langle t^*\rangle = 1$. In this case, eq. (79) coincides with the age model $T = t_\mathrm{b}(z) + L/\phi v_\mathrm{b}$ of the sediment. Above the critical size threshold

$$\frac{s}{s_0} = \psi_\mathrm{c} + \frac{v_\mathrm{b}}{D_\mathrm{s}\beta_0} \tag{80}$$

for which $v_\mathrm{t} \approx v_\mathrm{b}$, $\langle t^*\rangle$ increases very rapidly, leading to the occurrence of tracer particles that are much older than the sediment in which they are embedded. The size threshold is inversely related to $D_\mathrm{s}\beta_0/v_\mathrm{b}$ and is tendentially lower in cores with small sedimentation rates and strong bioturbation.

The relation between age offset, particle size, and sedimentation rate is clearly demonstrated by the difference between foraminifera and bulk sediment radiocarbon ages in two cores with similar water depths and different sedimentation rates (Thomson et al., 1995; Brown et al., 2001). In this example, the age offset of >150 μm foraminiferal calcite with respect to the bulk carbonate amounts to ~1.2 kyr in the slowly accumulating site ($v_\mathrm{b} \approx 3$ cm/kyr) and ~0.7 kyr in the faster accumulating site ($v_\mathrm{b} \approx 5.9$ cm/kyr).

Ferromanganese nodules

Ferromanganese nodules grow around cm-sized sees, such as sediment aggregates, organic remains, and rock fragments, at typical rates of 1–10 mm/Myr (von Stackelberg, 1997). Nodules are concentrated on the sediment surface and in the surface mixed layer, where they must remain much longer than regular sediment particles in order to reach the observed sizes, and only few are buried at larger depths (Finney et al., 1984). Nodule concentration profiles are thus characterized by a strong concentration gradient in SML, alike the steady-state segregation discussed above.

Various theories invoking bioturbation have been proposed as a qualitative explanation for the long permanence of ferromanganese nodules at the sediment surface, despite being denser than the underlying sediment (Piper and Fowler, 1980). Laboratory replica of the Brazil nut effect with mixtures of large, dense, and small, less dense particles suggest that denser nodules can be supported by the mixed sediment, as long as the nodule load does not exceed the sediment strength. The predominance of larger sizes among buried nodules and at the base of the mixed layer (Finney et al., 1984) suggests that there is a critical threshold of ~8 cm above which probability of sinking below the SML increases again. This threshold might also arise from the depth dependence of bioturbation, with the bottom of larger nodules reaching depths where the diffusion coefficient has decreased significantly. In the following, a simple nodule growth model is derived from the size segregation laws introduced in Section 3 and the stationary diffusion-advection model of Section 2.4.

Let $n_0(s)$ be the grain size distribution of seeds, with $n_0(s)\mathrm{d}s$ being the number of seeds with sizes comprised between s and $s+\mathrm{d}s$. Furthermore, let F_0 be the total flux of seeds at the sediment-water interface, in counts per unit of area and time, and $N(s,z)$ the number of nodules per unit of volume and of size at the depth z inside the SML, such that $N(s,z)\mathrm{d}s$ is the volume concentration of nodules having sizes comprised between s and $s+\mathrm{d}s$. It is assumed that all seeds develop a ferromanganese deposit that grows at a constant rate γ. Accordingly, the nodule size increases as $s(t)=s_0+\gamma t$ from a seed with size s_0. The growth and accumulation of nodules in the SML depends critically on the evolution of the burial probability in time. In case of constant seed supply and growth, vertical concentration profiles correspond to the stationary profiles calculated in Section 2.4, and can be expressed by $N(s,z)=n(s)r(z)$, where r is the normalized profile function, and $n(s)$ the nodule size distribution at the sediment-water interface. Mass conservation sets

$$0=\frac{\partial n(s)}{\partial t}=F_0 n_0(s)-\gamma L\frac{\partial n(s)}{\partial s}-v_\mathrm{b} r(s)n(s), \tag{81}$$

with $r=r(L)=R^{-1}$ being the inverse of the concentration ratio of stationary profiles described in Section 2.4. The three terms on the right-hand side of eq. (81) represent, in sequence: (1) the incoming flux of fresh seeds with size s, (2) the shift of the grain size distribution in time, due to particle growth, such that $n(s,t+\mathrm{d}t)=n(s-\gamma\mathrm{d}t,t)$, and (3) the outgoing flux at the bottom of the mixed layer. Rearrangement of terms in eq. (81) gives the ordinary differential equation

$$\frac{\mathrm{d}n(s)}{\mathrm{d}s}=\frac{1}{\gamma L}\left[F_0 n_0(s)-v_\mathrm{b} r(s)n(s)\right] \tag{82}$$

with general solution

$$n(s)=\frac{F_0}{\gamma L}\exp\left[-\frac{v_\mathrm{b}}{\gamma L}\int_0^s r(u)\mathrm{d}u\right]\left\{c+\int_0^s n_0(x)\exp\left[\frac{v_\mathrm{b}}{\gamma L}\int_0^x r(u)\mathrm{d}u\right]\mathrm{d}x\right\}, \tag{83}$$

where c is a constant. In the limit case of negligible seed growth $(\gamma \to 0)$ the grain size distribution of sees does not change, and the resulting condition $n \propto n_0$ is fulfilled only if $c = 0$ [4]. After rearranging the integration limits, the solution becomes

$$n(s) = \frac{F_0}{\gamma L} \int_0^s n_0(x) \exp\left[-\frac{v_\mathrm{b}}{\gamma L} \int_x^s r(u)\mathrm{d}u\right] \mathrm{d}x \,. \tag{84}$$

Grain size distributions are best expressed on a logarithmic scale, using $\mathfrak{n}(\ln s) = s n(s)$ and $\mathfrak{n}_0(\ln s) = s n_0(s)$, respectively. In this case, eq. (84) becomes

$$\mathfrak{n}(\xi) = \frac{F_0}{\gamma L} \int_{-\infty}^{\xi} \mathfrak{n}_0(x) \exp\left[\xi - \frac{v_\mathrm{b}}{\gamma L} \int_{e^x}^{e^\xi} r(u)\mathrm{d}u\right] \mathrm{d}x \,. \tag{85}$$

Numerical evaluations of eq. (85) for lognormal seed size distributions and values of R given by eq. (72–73) for the segregation velocity model of eq. (76–77) yield nodule grain size distributions that approach a constant plateau above a minimum size that depends on the typical seed size (Fig. S6a). This plateau can be explained by the continuous nucleation and growth of nodules with negligible sinking probability: in this case, the size of nodules is essentially proportional to their age. Real nodule size distributions are superiorly limited to 12–20 cm (Von Stackelberg, 1997). This limit can be explained by the fact that if nodules are prevented from sinking by bioturbation in the SML, their size cannot significantly exceed the SML thickness (Finney et al., 1984). Assuming that nodule support ceases as the center of mass sinks below the lower SML boundary, the maximum size is expected to be of the order of $2L$ or 10–30 cm, considering the typical range of L reported in the literature (Solan et al., 2019). The shape of the resulting nodule size distribution is strongly controlled by this maximum size, which might explain the range of distribution shapes observed in nodule fields with different sedimentation rates.

Seeds must have a minimum size to grow significantly and avoid burial. This is seen clearly by evaluating eq. (85) for lognormal size distributions of the incoming seeds, size segregation parameters obtained from the fit of tektite profiles in core MD90-0961, and sediment parameters representative for nodule fields. The mean bulk diffusion coefficient for sites with a water depth of ~4000 m is of the order of ~20 cm^2/kyr (Soetaert et al., 1996), with a mean

[4] The $\gamma \to 0$ limit

$$n(s) = \frac{F_0}{\gamma L} e^{-(\cdots)} \left[c + \int_0^s n_0(x) e^{(\cdots)} \mathrm{d}x\right]$$

of eq. (83) must be proportional to n_0. The exponential function inside the integral is growing fast, so that only x-values very close to the upper integration limit contribute to the result. In this case, $n_0(x) \approx n_0(s)$ and solution of the integral yields

$$n(s) = \frac{F_0}{\gamma L}\left[c\,e^{-(\cdots)} + n_0(s)\right].$$

This result is proportional to n_0 only if $c = 0$.

mixing depth of ~6 cm (Solan et al., 2019), and sedimentation rates comprised between 0.3 and 0.6 cm/kyr. In all cases, there is a finite number of seeds that grow enough to escape burial, which is roughly proportional to the fraction of incoming sees with sizes >2 mm (Fig. A6a). When this fraction raises above ~1%, the size distribution of the growing seeds contains a second contribution corresponding to ferromanganese nodules. The original seed distribution and the nodule distribution overlap at ~4 mm (Fig. A6b). The ~2 mm minimum seed size required to grow a nodule is comparable with the ~1 mm size of the smallest seeds observed inside recovered nodules (Von Stackelberg, 1997). A five-fold increase of the sedimentation rate suppresses the growth of nodules for all three incoming seed size distributions shown in Fig. 6b, in accordance with field observations. The same effect is obtained with a 43% decrease of the size segregation coupling parameter β_0, which couples the bulk diffusion coefficient with the segregation velocity.

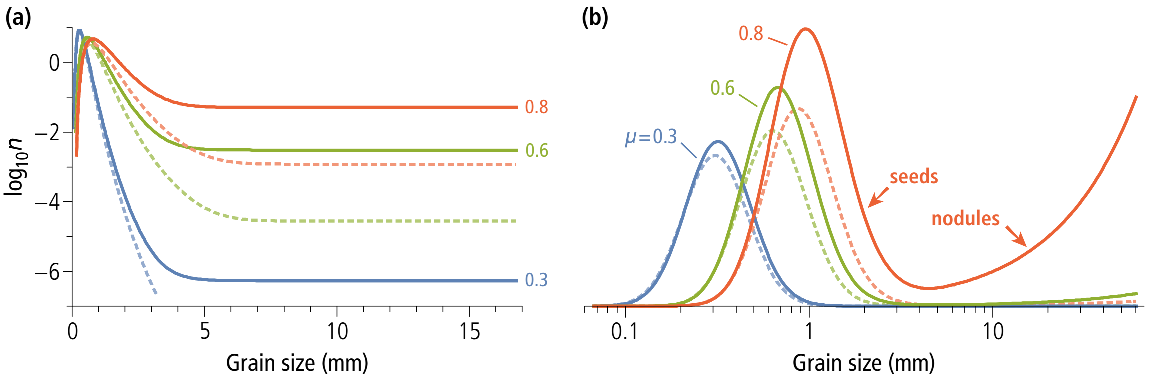

Figure A6. Steady-state distributions of seeds growing at a rate $\gamma = 5$ mm/Myr in a SML with the following properties: $D_s = 22$ cm^2/kyr, $L = 6$ cm, $s_0 = 13$ µm, and $v_b = 0.5$ cm/kyr, typical for ferromanganese nodule fields, and the size segregation parameters $\psi_c = 3$, $\beta_0 = 0.075$ m^{-1}, and $q = 0.252$ obtained from microtektite fits in core MD90-0961 (solid lines), represented on a linear (**a**) and a logarithmic (**b**) size scale. The three solid line curves correspond to incoming seeds with lognormal size distributions sharing the same logarithmic standard deviation of 0.4, and with logarithmic means of 0.3, 0.6, and 0.8 mm, respectively. The three dashed line curves correspond to the same incoming seeds, and a five-fold increase of the sedimentation rate ($v_b = 2.5$ cm/kyr), or, equivalently, a 43% decrease of the size segregation coupling constant ($\beta_0 = 0.0428$ m^{-1}).

Fitting tektite profiles

Fitting function

Tektite concentration profiles, in counts per unit of sediment mass, resulting from the homogeneous SML model described above, are given by

$$C_z(z) = \frac{\Phi}{\rho_s \phi_s} \frac{\eta\phi}{L} \mathcal{E}\left(\frac{\eta\phi}{L}(z_0 - z); G, \eta\right), \tag{86}$$

where Φ is the tektite fluence, in counts per unit of area, ρ_s the density of sediment particles, z_0 a reference depth corresponding roughly with the lowermost depth of tektite occurrence, ϕ_s the volume fraction of solids at z_0, $\phi \geq 1$ the ratio between ϕ_s at z_0 and in the SML, L the assumed thickness of the SML, $\mathcal{E}$ the probability density function of the non-dimensionalized escape time (eq. 51–53), and $\eta = 1$ for $v_t = v_b$ or $\eta = |1 - v_t/v_b|$ for $v_t \neq v_b$, with v_t being the segregation velocity of tektites and v_b the burial velocity in the SML. Volume concentrations, in counts per unit of sediment volume, are obtained by multiplying eq. (86) with the bulk sediment density $\rho_s \phi_s$. Examples of simulated tektite profiles obtained with eq. (86) are shown in Fig. A7. The shape of $\mathcal{E}$ depends on the inverse Péclet number G of the SML and on w. As discussed above, the underlying choice of a homogeneous SML has no effects on the shape of $\mathcal{E}$, since models with a different depth dependence of the diffusion coefficient D yield solutions that are precisely fitted by eq. (86) with proper choices of an equivalent SML thickness L and an equivalent diffusivity D.

For tektites of the same size class, the concentration profile of eq. (86) is controlled by the four aggregated parameters: (1) $\Phi/\rho_s \phi_s$ for the profile amplitude, (2) $\eta\phi/L$ for the profile width, (3) $\eta\phi z_0/L$ for the profile offset, and (4) $G = D/L\eta v_b$ for the profile shape. This means that some individual parameters need to be fixed using plausible values or additional measurements. For instance, ϕ_s, ρ_s, and ϕ are measurable physical properties of the sediment core, and v_b is fixed by the age model. This leaves the following optimizable parameters: (1) Φ for the profile amplitude, (2) L for the profile width, (3) z_0 for the profile position, and (4) D for the profile shape.

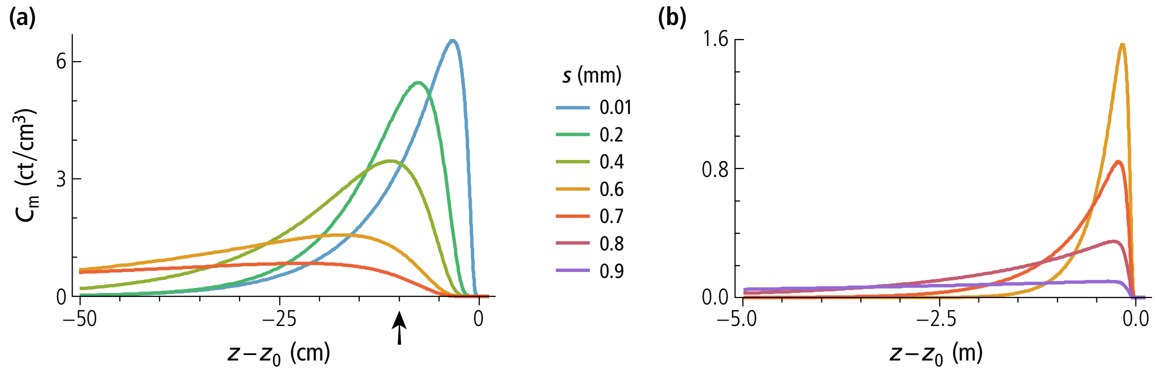

Figure A7. Simulated concentration profiles generated by an impulsive tracer input with the following parameters: $\rho_s = 2.3$ g/cm^3, $\phi_s = 0.6$, $\phi = 1$, $v_b = 3.8$ cm/kyr, $L = 10$ cm, $D = 30$ cm^2/kyr, $s_0 = 10$ µm, $\psi_c = 3$, $q = 0.5$, $\beta_0 = 0.2$ m^{-1}, $\Phi = 100$ ct/cm^2, and selected tracer particle sizes s in mm. The corresponding segregation velocities range from $v_t = 0$ for $s \leq 0.03$ mm to $v_t = 5.4$ cm/kyr for $s = 0.9$ mm. **(a)** Tracer profiles within 50 cm from z_0: the event horizon, defined as the stratigraphic depth where the tracer would be found without bioturbation, is marked by an arrow. **(a)** Tracer profiles within 5 m from z_0. Notice how tracer particles with $v_t \geq v_b$ are homogeneously spread in the sediment column.

The remaining parameter η cannot be determined without additional constraints. Because η is a function of the segregation velocity v_t, the required additional constraint is provided by the fit of concentration profiles corresponding to different segregation velocities and thus size classes, including smaller sizes for which $v_t = 0$ and therefore $\eta = 1$, using suitable models for the grain size dependence of D and v_t. A grain size class with size limits $[s_1, s_2]$ is then fitted by

$$C_z(z) = \frac{\Phi}{\rho_s \phi_s} \frac{\phi}{L} \int_{s_1}^{s_2} g_t(s) \eta \mathcal{E}\left(\frac{\eta\phi}{L}(z_0 - z); G, w \right) \mathrm{d}s, \qquad (87)$$

where $g_t(s)$ is the empirical tektite size distribution, obtained for instance from total counts.

Poisson regression

The primary measured quantity in case of discrete tracer particles, such as tektites, is particle counts per unit of sediment mass. Model profiles must therefore be fitted using Poisson regression to correctly account for skewness of Poisson error distributions. For this purpose, consider a set of counts n_{kl} for tracers belonging to size intervals $\mathbf{s}_l = [s_l^{\min}, s_l^{\max}]$ obtained from samples with masses m_k taken at different depths. The empirical grain size distribution of the tracer is given by the total concentrations

$$S_l = \frac{\sum_k n_{kl}}{\sum_k m_k} \qquad (88)$$

of each grain size class l. This discrete distribution is fitted using a model function $g_t(s)$ with unit integral, such that

$$S_l = S_0 \int_{\mathbf{s}_l} g_t(s) \mathrm{d}s, \qquad (89)$$

where S_0 is a normalization constant obtained from the fit, with no further use. The counts n_{kl} yields estimates $c_{kl} = n_{kl}/m_k$ of the l-th size class concentration at various depths in the sediment column. These estimates need to be compared with modeled concentration profiles given by $C_z(z_k, \mathbf{s}_l; \boldsymbol{\theta})$ in eq. (88), where $\boldsymbol{\theta} = (\Phi, z_0, L, D_s, q, \beta_0)$ is the vector formed by the unknown parameters Φ (the total fluence for all sizes), z_0 (the reference depth), L (the thickness of the surface mixed layer), D_s (the diffusion coefficient of sediment particles), q (the power exponent for the tracer diffusivity), and β_0 (the coupling constant between diffusivity and grain size segregation).

The maximum likelihood (ML) model of the measured concentrations is obtained by maximizing the probability of obtaining the measured counts n_{kl} from random realizations of Poisson processes with corresponding (unknown) rates $\lambda_{kl} = m_k C_z(z_k, \mathbf{s}_l; \boldsymbol{\theta})$. Accordingly, tektite counts are described by the Poisson distribution

$$\mathcal{P}(n,\lambda)=\frac{\lambda^n e^{-\lambda}}{n!}. \tag{90}$$

The validity of this approach is limited only by the assumption that the bioturbation process is homogeneous at the sample scale, so that the randomness of the counts n_{kl} is entirely controlled by Poisson statistics. Maximization of the joint probability of all realizations n_{kl} is equivalent to the minimization of

$$-\sum_{k,l}\ln\mathcal{P}(n_{kl},\lambda_{kl})=-\sum_{k,l}n_{kl}\ln\lambda_{kl}-\lambda_{kl}-\ln n_{kl}!. \tag{91}$$

Since the last term in eq. (91) is independent of the model parameters, it is sufficient to minimize the negative log-likelihood

$$-\mathscr{L}(\boldsymbol{\theta})=\sum_{k,l}m_k C(z_{kl},\mathbf{s}_l;\boldsymbol{\theta})-n_{kl}\ln m_k C(z_{kl},\mathbf{s}_l;\boldsymbol{\theta}) \tag{92}$$

with respect to the model parameters in $\boldsymbol{\theta}$. One problem with the use of eq. (92) is that non-numerical outputs are generated when C is exactly zero, that is, before the event onset. The inclusion of zero counts before the event onset, however, is essential for constraining the time range. Therefore, $\mathscr{L}$ needs to be regularized by assigning a small, non-zero lower limit λ_0 for the Poisson rate, so that

$$-\mathscr{L}(\boldsymbol{\theta})=\sum_{k,l}\max\left[\lambda_0,m_k C(z_{kl},\mathbf{s}_l;\boldsymbol{\theta})\right]-n_{kl}\ln\max\left[\lambda_0,m_k C(z_{kl},\mathbf{s}_l;\boldsymbol{\theta})\right]. \tag{93}$$

The minimum rate λ_0 can be interpreted as the probability of "spurious" counts, due for instance to misattribution, contamination, or other error sources.

As discussed above, the definition of C though the eigenfunction expansion of the escape time distribution $\mathcal{E}$ is computationally expensive, which means that the minimization of eq. (94) in the six dimensions spanned by the model parameters should be based on as few evaluations of C as possible, avoiding the explicit use of derivatives. Suitable algorithms include stabilized forms of the two-point gradient descent method, as used in this paper.

Bayesian uncertainty estimates and statistical tests

The effects of counting uncertainties on model results can be estimated with Monte Carlo simulations. For this purpose, actual counts are replaced by simulated ones, based on empirical probability distributions. For the derivation of such distributions, consider first the case of N realizations $\mathbf{n}=(n_1,n_2,\ldots,n_N)$ of a Poisson variate n with rate λ. The corresponding probability function is

$$p(n\,|\,\lambda)=\mathcal{P}(n;\lambda)=\frac{\lambda^n e^{-\lambda}}{n!} \tag{94}$$

for any integer $n \geq 0$. In Bayesian theory, the unknown rate $\lambda > 0$ is itself a random variate for which a prior probability distribution $p_{\text{prior}}(\lambda)$ is assumed. This distribution might reflect some physical constraints, but it is mostly unknown. In this case, the most unassuming choice of p_{prior} is a uniform distribution over $(0,\infty)$. The corresponding unnormalized posterior distribution

$$p_{\text{post}}(\lambda|\mathbf{n}) \propto p(\mathbf{n}|\lambda)p_{\text{prior}}(\lambda) \propto \lambda^{n_1+n_2+\ldots}e^{-N\lambda} \tag{95}$$

of λ for a given $\mathbf{n}$ is proportional to the Gamma distribution

$$\Gamma(\lambda;a,b) = \frac{b^a}{\Gamma(a)}\lambda^{a-1}e^{-bx}, \tag{96}$$

with $a = 1 + n_1 + n_2 + \ldots + n_N$, and $b = N$. This result can be generalized to the case where p_{prior} is a Gamma distribution with parameters (a_0, b_0). The corresponding posterior distribution is also a Gamma one, with parameters $a' = a_0 + a$ and $b' = b_0 + b$. For this reason, the Gamma distribution is the conjugate prior of the Poisson distribution. The posterior parameters a' and b' become increasingly dominated by the data as the size of $\mathbf{n}$ increases. Monte Carlo simulations require the generation of simulated counts according to the so-called posterior predictive distribution

$$p_{\text{pred}}(n|\mathbf{n}) = \int_0^\infty \mathcal{P}(n|\lambda)p_{\text{post}}(\lambda|\mathbf{n})\mathrm{d}\lambda. \tag{97}$$

Using the Gamma distribution as $p_{\text{post}}(\lambda)$, eq. (97) yields the negative binomial distribution

$$\mathcal{B}_-(n;a',(b'+1)^{-1}) = \frac{\Gamma(n+a')}{n!\Gamma(a')}\left(\frac{b'}{b'+1}\right)^{a'}\left(\frac{1}{b'+1}\right)^n \tag{98}$$

for all integers $n \geq 0$.

In the context of tektite profiles, there is one sample with count n_i for each depth and each size class, whereby all counts are assumed to be independent from each other. Each count is the random realization of a Poisson variate with rate λ_i. The posterior distribution of λ_i obtained from a uniform prior is a Gamma distribution with parameters $a_i' = n_i$ and $b_i' = 1$. The corresponding posterior predictive distribution is thus $\mathcal{B}_-(n;n_i,1/2)$. If $n_i = 0$, the Gamma distribution is evaluated for the limit $a_i' \to 0$. Taking the limit of this $\mathcal{B}_-$ for $n_i = 0$ yields a probability of 1 for $n = 0$ and 0 for $n > 0$. The simulated Monte Carlo simulations $\tilde{n}_i$ of n_i are therefore random variates generated by $\mathcal{B}_-(\tilde{n};n_i,1/2)$.

The significance of grain size segregation effects predicted by the bioturbation model is evaluated by testing the hypothesis H_1 with free parameters $\theta_1 = (\Phi, z_0, L, D_s, q, \beta_0)$ against the following null hypotheses H_0: (1) diffusion is grain size-dependent ($q > 0$), but size segregation does not occur ($\beta_0 = 0$), as in Wheatcroft (1992), and (2) diffusion and advection are

independent of size ($q=0,\ \beta_0=0$). The goodness of H_1 with respect to H_0 is quantified by the likelihood ratio

$$\Lambda = \frac{\mathscr{L}(\theta_0|\mathbf{c})}{\mathscr{L}(\theta_1|\mathbf{c})} \tag{99}$$

where $\mathbf{c}=(c_1,c_2,\ldots)$ is the vector of all concentrations, and θ_0 is the vector of free parameters in H_0. The smaller is Λ, the higher is the probability that H_1 is a better model than H_0. Accordingly, the null hypothesis that the two models are equivalent descriptions of the data within the error range is rejected if Λ is small than a certain critical threshold. The Wilks theorem states that, as the sample size approaches infinity, the test statistics $-2\ln\Lambda$ approaches asymptotically a random variate with a chi-squared distribution χ_ν^2 with degrees of freedom ν given by the difference in the dimensionality of θ_1 and θ_0. The rejection limit of the test statistics at a confidence level p_0 is thus obtained from the solution of $F(x,\nu)=p_0$ with respect to x, where

$$F(x,\nu)=1-\frac{\Gamma(\nu/2,x/2)}{\Gamma(\nu/2)} \tag{100}$$

is the cumulative distribution of χ_ν^2, with Γ being the (incomplete) gamma function. The limit for rejecting the null hypothesis of no grain size segregation $(\nu=1)$ at a 95% level is 3.8415, while the limit for rejecting the null hypothesis of no grain size effects $(\nu=2)$ at a 95% level is 5.9915. The same limits at a 99% confidence level are 6.6349 and 9.2103, respectively.

Equivalency of diffusivity profiles

First-principles considerations about the consumption of organic matter by benthic organisms, the depth distribution of such organisms and their burrowing traces, and the depth distribution of tracers (Fornes et al., 2001), suggest that the long-term average sediment mixing rate is a monotonically decreasing function of depth below the sediment-water interface. In simple terms, it can be assumed that the mixing rate, and thus the bulk diffusion coefficient D_s of diffusive models, is proportional to the total biomass, which is in turn proportional to the product of available organic matter and its consumption rate. A general expression for the depth dependence of D_s can be obtained by assuming $D_s=bC_{org}$ to be proportional to the concentration C_{org} of organic matter, whose consumption obeys a first-order kinetic reaction with rate k_{org}. The steady-state diffusion-advection equation for constant organic carbon input and negligible burial velocity is then given by

$$\frac{\partial}{\partial z}\left[bC_{org}\frac{\partial C_{org}}{\partial z}\right]-k_{org}C_{org}=0 \tag{101}$$

with solution $C_{org}\propto(1-z/L)^2$ for $z\le L$, where L is the depth at which organic matter is completely consumed. More realistic models must take the quality of organic matter into consideration, with fresh organic material being consumed more readily than older stocks. In

this case, eq. (101) is replaced by multi-pool models consisting of a set of equations of the same type, one for each pool of organic matter with specific consumption rate. These models yield exponential-like profiles of total organic carbon. Because fresher organic matter is more readily consumed, its concentration is higher near the sediment-water interface, where it sustains a larger benthic organism community. Accordingly, the apparent bulk SML diffusivity obtained from younger tracers, such as chlorophyll-a and short-lived radioactive isotopes, is larger than that of older tracers (Fornes et al., 2001), and the apparent SML thickness is smaller (Fig. A8).

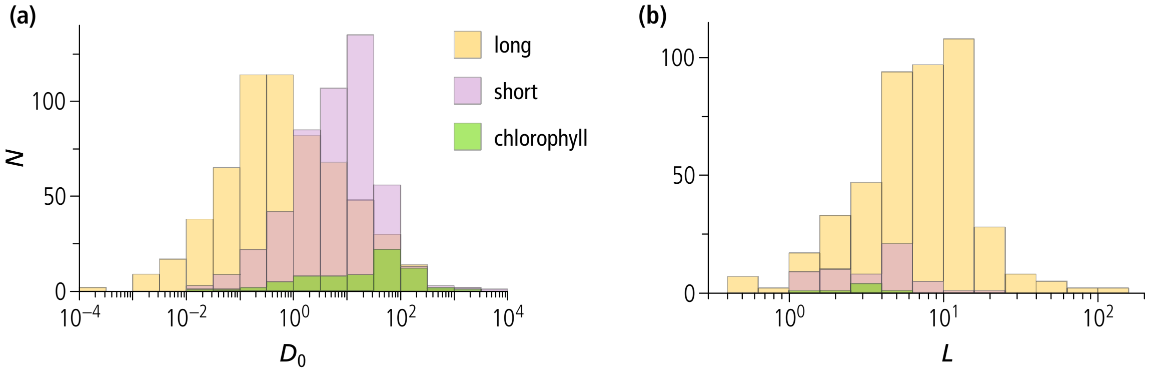

Figure A8. Apparent diffusivity (**a**) and thickness (**b**) of homogeneous surface mixed layers deduced from stable tracers or long-lived radioisotopes with half-lives >22 years (^{210}Pb, ^{137}Cs, ^{32}Si, $^{239/240}$Pu, glass beads, tektites), short-lived radioisotopes (^{234}Th, ^{235}Th, ^{228}Th, ^{7}Be), and chlorophyll-a. Data from a worldwide compilation by Solan et al. (2019).

In principle, organic matter degradation continues at very large depths, due to the slow bacterial degradation of the refractory component; however, the high energy requirements of locomotion in increasingly compacted sediment limits the occurrence of motile organisms that produce significant mixing to a surface layer with finite thickness $L_{\max}$. The depth dependence of the diffusion coefficient is thus conveniently expressed by a pseudo-exponential function that becomes exactly zero below $L_{\max}$. In mathematical terms, $D_s(z)=D_0 y(z/L)$, where D_0 is the maximum diffusivity at the sediment-water interface, $L<L_{\max}$ the typical length scale of the mixed layer, and y a monotonically decreasing function with $y(0)=1$ and $y(x)=0$ for $x \geq x_0$ with $x_0=L_{\max}/L$. For instance, an exponential diffusivity profile is represented by $y(x)=\Theta(x/x_0)e^{-x}$, where Θ is a function representing a smooth transition from 1 to 0 for $x \leq x_0$. In case of negligible compaction inside the mixed layer, the diffusion-advection equation for the concentration C of tracer particles with diffusivity $D_t=\alpha D_s$ and segregation velocity $v_t=\beta D_s$ (see Section 3) is given by

$$\frac{\partial C}{\partial t}=\frac{\partial}{\partial z}D_t\frac{\partial C}{\partial z}+(v_t-v_b)\frac{\partial C}{\partial z} \tag{102}$$

with boundary condition

$$\left[(v_b - v_t)C - D_t \frac{\partial C}{\partial z}\right]_{z=0} = F(t), \tag{103}$$

at the sediment-water interface, controlled by the incoming tracer flux F. Unlike the case of the homogeneous SML discussed in Section 3, a second boundary condition is not needed, because $D_t = v_t = 0$ at $z = L_{max}$, so that the tracer flux just above L_{max} is already equal to the flux $v_b C$ in the historical layer.

General solutions of eq. (102–103) are not known, so that their use for modeling real concentration profiles is impractical. However, a well-defined relationship exists between numerical solutions and the case of a homogeneous mixed layer, so that concentration profiles can be still modeled using the solutions obtained in Section 3. As a first step, the same variable substitutions $x = z/L$ and $t^* = v_b t/L$ are used to convert eq. (102) into the scaled form

$$\frac{\partial C}{\partial t^*} = \frac{\partial}{\partial x}\left[\alpha G_0 y(x) \frac{\partial C}{\partial x} + \big(\beta L G_0 y(x) - 1\big)C\right], \tag{104}$$

with $G_0 = D_t(0)/L v_b$ being the inverse Péclet number of regular sediment particles at the sediment-water interface. The corresponding boundary condition for calculating the impulse response is

$$\left[(1 - \beta L G_0)C - \alpha G_0 \frac{\partial C}{\partial x}\right]_{x=0} = \delta(t^*). \tag{105}$$

Eq. (104–105) depend on two parameters, αG_0 and $\beta L G_0$, which control the strength of scaled diffusion and segregation velocity, respectively. In case of tracer particles behaving like the bulk sediment ($\alpha = 1$ and $\beta = 0$), the resulting equations

$$\frac{\partial C}{\partial t^*} = \frac{\partial}{\partial x}\left[G_0 y(x) \frac{\partial C}{\partial x} - C\right] \tag{106}$$

and

$$\left[C - G_0 \frac{\partial C}{\partial x}\right]_{x=0} = \delta(t^*) \tag{107}$$

depend on a single parameter, G_0, and the shape of y. Numerical solutions for a pseudo-exponential diffusivity profile $y(x) = \Theta(x_0 - x)e^{-x}$ with $x_0 = 15$ and

$$\Theta(x) = \frac{1}{2}\begin{cases} 0 & , x < 0 \\ 1 - \cos(\pi x/2) & , x < 2 \\ 2 & , \text{else} \end{cases} \tag{108}$$

differ from those of a homogeneous SML in the way they depend on G_0 (Fig. A9). The impulse response of a homogeneous SML varies from a narrow, Gaussian-like peak around the mean escape time $\langle t^* \rangle$ for $G_0 \ll 1$ to a one-sided exponential for $G_0 \gg 1$, with a sharp left limit given by $\langle t^* \rangle - 1 = 0$. This limit corresponds to the instantaneous transport of tracer particles

to the bottom of the SML when diffusion dominates over advection. Exponential diffusivity profiles produce impulse responses that become increasingly wide as diffusion increases over advection, without approaching a sharp left limit as for a homogenous SML. This can be explained by the fact the depth at which the burial velocity becomes dominant is always equal to L in the case of a homogeneous mixed layer, while it depends on G_0 in the case of an exponential diffusivity profile.

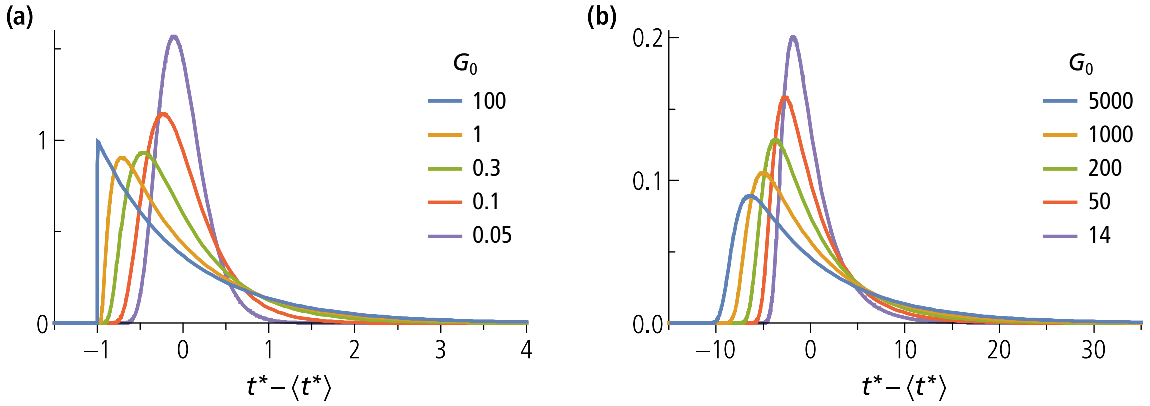

Figure A9. Nondimensionalized impulse responses for a homogeneous mixed layer (**a**) and a mixed layer with exponentially decaying diffusivity (**b**), for $v_t = 0$ and selected values of the inverse Péclet number G_0. In both panels, $t^* = v_b t / L$ is the dimensionless time and $\langle t^* \rangle$ the corresponding expectation, with L being the thickness of the mixed layer in (a), and the depth at which the diffusivity is $1/e \approx 0.368$ times the value at the sediment-water interface in (b).

Despite all differences, the impulse response $\mathcal{E}_{\text{exp}}(t^* - \langle t^* \rangle, G_0)$ generated by an exponential diffusivity profile with inverse Péclet number G_0 is almost perfectly matched by a rescaled version

$$\mathcal{E}'_{\text{exp}}(t^*) = \frac{1}{\omega} \mathcal{E}\left(\frac{t^* - \langle t^* \rangle}{\omega}, \varkappa G_0 \right) \tag{109}$$

of the impulse response of a homogeneous SML with a smaller inverse Péclet number $\varkappa G_0$, where $0 < \varkappa < 1$, and with the dimensionless time axis stretched by a factor ω (Fig. A10a). The almost perfect match between the two mixed layer models means that impulsive tracer concentration profiles can be fitted equally well by both models. Since SMLs with constant and with exponential diffusivity profiles represents extreme cases of postulated bioturbation models, the comparisons in Fig. A10a show that it is intrinsically impossible to resolve the depth dependence of sediment mixing on the basis of stable tracer profiles.

The relation between models based on constant and exponential diffusivity profiles can be understood by comparing the tracer concentration profiles obtained from the corresponding impulse responses. If follows from eq. (77) and $\eta = 1$ from the rescaling used to obtain eq. (105) that

$$C_z(z,T)=\frac{\Phi}{\rho_s\phi_s}\frac{\phi}{L}\mathcal{E}_{\exp}\left(\frac{\phi v_b}{L}(T-t_b(z)),G_0\right) \tag{110}$$

is the concentration profile of tracer particles deposited at time T on a sediment with exponential diffusivity profile with inverse Péclet number G_0 at $z=0$.

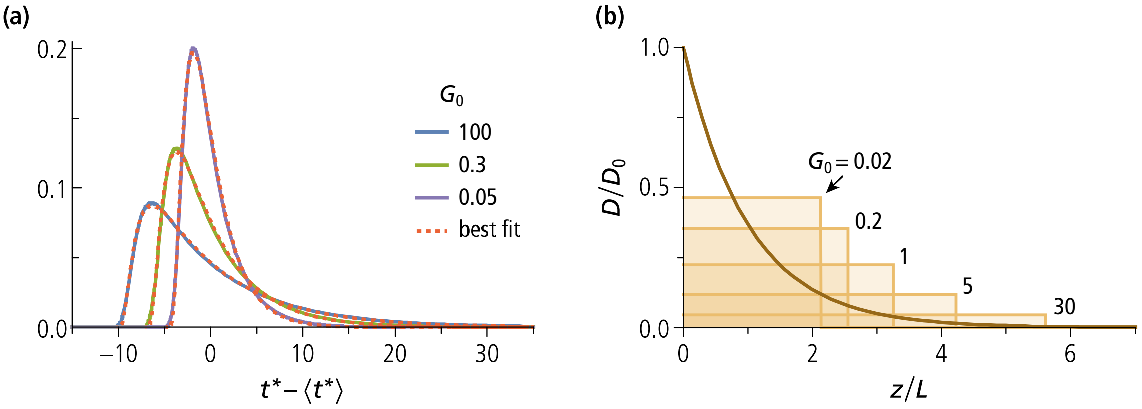

Figure A10. (**a**) Nondimensionalized impulse responses for a SML with $v_t=0$ and exponentially decaying diffusivity (solid lines), compared to least-squares fits with rescaled impulse responses from a homogeneous SML, according to eq. (109) (dashed lines). The rescaling parameters are $(\varkappa,\omega)=(0.322, 6.653)$ for $G_0=100$, $(\varkappa,\omega)=(0.0360, 2.687)$ for $G_0=0.3$, and $(\varkappa, \omega)=(0.0986, 2.214)$ for $G_0=0.05$. (**b**) Exponential diffusivity profile (brown line) and diffusivity profiles of equivalent homogeneous SMLs (shaded) for selected values of G_0.

The other parameters in eq. (110) are the tracer fluence Φ, the time t_b required for a particle to be buried from the maximum bioturbation depth L_{max} to z, and $\phi=\phi_s(z)/\phi_s(L_{max})$. For comparison, a homogeneous mixed layer of thickness ωL and inverse Péclet number $\varkappa G_0$ produces the equivalent profile

$$C_z(z,T)=\frac{\Phi}{\rho_s\phi_s}\frac{\phi}{\omega\eta L}\mathcal{E}\left(\frac{\phi v_b}{\omega\eta L}(T-t_b(z)),\varkappa G_0\right), \tag{111}$$

with $\eta=1$ for $v_t=v_b$ or $\eta=|1-v_t/v_b|^{-1}$ for $v_t\neq v_b$. Accordingly, a SML with exponential diffusivity profile $D_s=D_0e^{-z/L}$ is equivalent to a homogeneous SML with thickness ωL and diffusivity $\varkappa\omega D_0$, with ω and $\varkappa$ being functions of $G_0=D_0/Lv_b$. In the limit case $G_0\to 0$ where advection is dominant, the equivalent homogeneous SML has a thickness $2L$ and a diffusion coefficient $D_0/2$ (Fig. A10b). As G_0 increases, the equivalent homogeneous SML becomes thicker, and its diffusivity decreases. There is no upper limit to this trend. The equivalency between exponential and homogeneous SMLs is expressed empirically by the diffusivity ratio

$$\frac{D^{\text{hom}}}{D_0^{\text{exp}}}=\frac{1}{2}\left[1+2.692\,G_0^{0.7544}+0.2722\,G_0^{1.3723}\right]^{-0.5748} \tag{112}$$

and the ratio

$$\frac{L^{\text{hom}}}{L^{\text{exp}}} = 2 + 1.544 \ln\left[1 + 1.182\, G_0^{0.611}\right] \tag{113}$$

between length scales (Fig. A11), where the superscripts "hom" and "exp" refer to the homogeneous SML and the SML with exponential diffusivity profile, respectively.

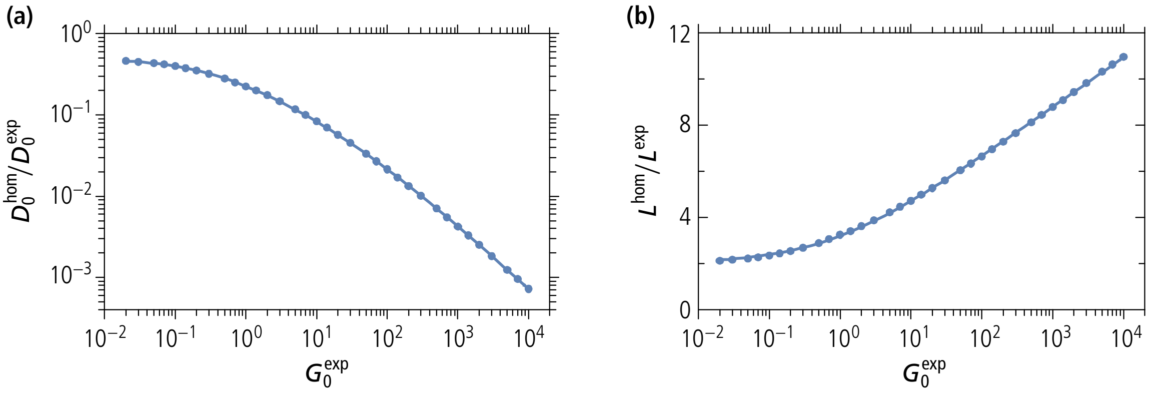

Figure A11. Diffusivity (**a**) and thickness (**b**) of a homogeneous mixed layer producing the same impulse response as a mixed layer with exponential diffusivity profile. Dots represent best-fit parameters obtained by fitting numerical solutions of eq. (106–107) with the impulse response of a homogeneous mixed layer. Lines are given by eq. (112–113).

These equations can be used to fit concentration profiles from a supposedly exponential diffusivity profile in two steps: in the first step, a fit is obtained with the analytical solution for a homogeneous SML, for which a suitable analytical approximation exists (e.g., eq. 86). The parameters of the exponential diffusivity model are then obtained by solving eq. (112–113) with respect to D^{exp} and L^{exp}, using $G_0 = D_0^{\text{exp}}/(L^{\text{exp}} v_\text{b})$.

6. Table of symbols

Symbol	Unit	Definition
a,b	—	Parameters of the Gamma distribution
$\mathcal{A}$	1/kyr	Age distribution of a tracer at given depth below the SML
$\mathcal{B}_-$	—	Negative binomial distribution
c_{kl}	ct/g	Concentration of size class l tektites in sample k
C	(g or ct)/g	Mass concentration of a tracer
C_v	(g or ct)/cm^3	Volume concentration of a tracer
C_z	(g or ct)/g	Mass concentration of a tracer below the SML (tracer profile)
D_s	cm^2/kyr	Self-diffusion coefficient of regular-sized sediment particles
D_t	cm^2/kyr	Diffusion coefficient of a tracer
D_0	cm^2/kyr	D_s at the sediment-water interface
E	1/kyr	Probability density function of the escape time t_L
$\mathcal{E}$	—	Probability density function of t_L^* (nondimensionalized)
$\mathcal{E}_\mathrm{exp}$	—	$\mathcal{E}$ for a SML with exponential diffusivity profile
F	(g or ct)/cm^{2}kyr	Incoming tracer flux
F_s	g/cm^{2}kyr	Incoming sediment flux
F_0	ct/cm^{2}kyr	Incoming flux of ferromanganese nodule seeds
g_t	1/mm	Modeled grain size distribution of tektites
G	—	Generic Péclet number
G_s	—	Inverse Péclet number for the bulk sediment
G_0	—	Inverse Péclet number at the sediment-water interface
$\mathcal{G}$	—	Solution of the nondimensionalized diffusion-advection equation
H_0,H_1	—	Testing hypotheses
$\mathfrak{I}$	1/kyr	Impulse response in the time domain
J_s	cm/kyr	Volumetric flux of the bulk sediment
L	cm	Thickness of a homogeneous SML, bioturbation depth
L_max	cm	Maximum mixing depth in non-homogeneous SML
$\mathscr{L}$	—	Log-likelihood
m_{kl}	g	Mass of sample k used for tektite counts
n	ct/cm^3	Volumetric concentration of ferromanganese nodules at $z=0$
n_{kl}	ct	Tektite counts in sample k for grain size class l
n_0	1/cm	Size distribution of ferromanganese nodule seeds
N	ct/mm	Volumetric concentration of ferromanganese nodules
p	—	Probability
$\mathcal{P}$	—	Poisson distribution
Pe	—	Péclet number (advection-to-diffusion ratio)
q	—	Power law exponent for the grain size dependence of D_t
r	—	Normalized stationary tracer profile
R	—	Ratio between tracer concentration at $z=0$ and given depth
s	mm	Particle size
s_0	mm	Typical sediment grain size (mean or median), or seed size
$\mathbf{s}_l$	mm	l-th tektite grain size class
S_l	ct/g	Empirical grain size distribution for size class l
$\mathcal{S}$	—	Nondimensionalized survival probability
t	kyr	Time

t^*	—	Nondimensionalized time
t_L	kyr	Escape time from the SML
t_L^*	—	Nondimensionalized escape time from the SML
T	kyr	Age
T_L	kyr	Advective escape time (deterministic part of t_L)
t_b	kyr	Time required for burial to a given depth below the SML
v_a	cm/kyr	Advective transport velocity in the SML
v_b	cm/kyr	Burial velocity of regular sediment particles
v_s	cm/kyr	Velocity of particles relative to the sediment-water interface
v_t	cm/kyr	Upward velocity of a tracer relative to regular sediment particles
w	—	Normalized absolute burial velocity discontinuity at $z = L$
x	—	Nondimensional depth in sediment
y	—	Function expressing the depth dependence of D_s
z	cm	Depth below the sediment-water interface
z_0	cm	Reference depth, location of depth profile
z_k	cm	Depth of the k-th sample used for tektite counting
α	—	Coefficient for the grain size dependence of D_t
β	1/cm	Coupling coefficient between D_s and v_t
β_0	1/cm	Coupling coefficient between D_s and ψ
γ	mm/Myr	Ferromanganese nodule grow rate
Γ	—	Gamma distribution
δ	—	Dirac impulse function
ζ	cm	Burial depth, as inverse function of t_b
η	—	Correction factor for time scaling
θ	—	Parameter vector for tektite profile fits
Θ	—	Smooth step function
$\varkappa$	—	Rescaling factor for G_0 to match different SML
λ	—	Eigenvalue or rate of a Poisson process
λ_{kl}	—	Poisson rate for the tektite size class l in sample k
λ_0	—	Minimum Poisson rate associable with spurious counts
Λ	—	Log-likelihood ratio
ν	—	Degrees of freedom in a model
ρ_s	g/cm^3	Density of sediment particles
ρ_t	g/cm^3	Density of a tracer
ϕ_s	—	Volume fraction of solids
ϕ_t	—	Volume fraction of a tracer
Φ	(g or ct)/cm^2	Tracer fluence (referred to an impulsive input)
χ_ν^2	—	Chi-squared distribution with ν degrees of freedom
ψ	—	Tracer-to-sediment size ratio
ψ_c	—	Critical value of ψ above which segregation takes place
ω	—	Rescaling factor for t^* to match different SML
